# The rapid proximity labeling system PhastID identifies ATP6AP1 as an unconventional GEF for Rheb

**DOI:** 10.1038/s41422-024-00938-z

**Published:** 2024-03-06

**Authors:** Ran Feng, Feng Liu, Ruofei Li, Zhifen Zhou, Zhuoheng Lin, Song Lin, Shengcheng Deng, Yingying Li, Baoting Nong, Ying Xia, Zhiyi Li, Xiaoqin Zhong, Shuhan Yang, Gang Wan, Wenbin Ma, Su Wu, Zhou Songyang

**Affiliations:** 1 MOE Key Laboratory of Gene Function and Regulation, State Key Laboratory of Biocontrol, Guangzhou Key Laboratory of Healthy Aging Research, School of Life Sciences, Institute of Healthy Aging Research, Sun Yat-sen University, Guangzhou, Guangdong China; 2 Sun Yat-sen Memorial Hospital, Sun Yat-sen University, Guangzhou, Guangdong China

**Keywords:** Insulin signalling, TOR signalling, Proteomic analysis

## Abstract

Rheb is a small G protein that functions as the direct activator of the mechanistic target of rapamycin complex 1 (mTORC1) to coordinate signaling cascades in response to nutrients and growth factors. Despite extensive studies, the guanine nucleotide exchange factor (GEF) that directly activates Rheb remains unclear, at least in part due to the dynamic and transient nature of protein–protein interactions (PPIs) that are the hallmarks of signal transduction. Here, we report the development of a rapid and robust proximity labeling system named *Pyrococcus horikoshii* biotin protein ligase (*Ph*BPL)-assisted biotin identification (PhastID) and detail the insulin-stimulated changes in Rheb-proximity protein networks that were identified using PhastID. In particular, we found that the lysosomal V-ATPase subunit ATP6AP1 could dynamically interact with Rheb. ATP6AP1 could directly bind to Rheb through its last 12 amino acids and utilizes a tri-aspartate motif in its highly conserved C-tail to enhance Rheb GTP loading. In fact, targeting the ATP6AP1 C-tail could block Rheb activation and inhibit cancer cell proliferation and migration. Our findings highlight the versatility of PhastID in mapping transient PPIs in live cells, reveal ATP6AP1’s role as an unconventional GEF for Rheb, and underscore the importance of ATP6AP1 in integrating mTORC1 activation signals through Rheb, filling in the missing link in Rheb/mTORC1 activation.

## Introduction

The mechanistic target of rapamycin (mTOR)^1–7^ pathway integrates internal and external signals such as changes in nutrients,^8–23^ energy,^24,25^ and growth factors^24,26–31^ to control cell growth and metabolism.^1,32–40^ Dysregulation of mTOR signaling can lead to metabolic disorders,^38,41^ neurodegeneration,^42^ cancer,^43–48^ and aging.^49–52^ As a key hub that balances anabolic and catabolic processes,^38,53–56^ mTOR complex 1 (mTORC1) regulates metabolism,^38,41,57,58^ mRNA translation,^59–61^ and autophagy^62–65^ through phosphorylation of multiple substrates.^66–73^ Over the past three decades many studies have been devoted to elucidating mTORC1 function and regulation.^21,74,75^ It has been shown that Ras-related GTP-binding proteins (Rags)^8,9^ and Ras homolog enriched in the brain (Rheb),^24,28,76,77^ whose nucleotide-loading states are modulated by GTPase-activating proteins (GAPs) and guanine nucleotide exchange factors (GEFs),^78^ act as signal switches for mTORC1-mediated anabolism when amino acids, growth factors, and energy are readily available.^42^ Activated Rags and Rheb are central to the activation of mTORC1 signaling. Mammalian mTORC1 has been shown to be recruited by the activated Rag heterodimers to the lysosomal membrane,^10,11,79–82^ where it meets Rheb. Rags are tethered to the lysosomal membrane through the Ragulator complex, which also acts as the GEF for RagA/B.^83^ Growth factors such as insulin can stimulate Rheb activity. For instance, insulin treatment can lead to phosphorylation of the Rheb GAP (the TSC complex) by Akt,^24,26,28,84,85^ thus releasing TSC from lysosomes and freeing Rheb. Activated GTP-bound Rheb can then directly stimulate the kinase activity of mTORC1.^24,27,30,86^ Therefore, the conversion of Rheb from the inactive GDP-bound state to the active GTP-bound state is essential for mTORC1 activation and function. However, the factors/proteins that act as Rheb GEF(s) remain controversial.^32,87–89^

Transient protein–protein interactions (PPIs) are necessary and essential to signaling cascades such as Rheb-mTORC1, especially the early events that govern Rheb-mTORC1 activation, but they are also more difficult to pinpoint and study. In the past few years, several platforms that enable PPI network identification in live cells have been developed. In particular, proximity-dependent biotin identification (BioID), which relies on mutated bacterial biotin protein ligases (BPLs) (e.g., BirA), has proven highly efficient.^90^ However, slow labeling kinetics of BioID and BioID2 (16–24 h) makes it difficult to differentiate between transient and stable PPIs.^91^ Both TurboID^92^ and APEX2^93^ can label interacting proteins within 1 min, but high cytotoxicity has limited their applications.^94^ Consequently, a promiscuous biotin ligase that is compact, catalytically efficient, and well-tolerated by cells is needed to better capture transient regulatory interactions that are at the heart of signaling cascades. To this end, we developed a novel BioID system termed *Pyrococcus horikoshii* BPL (*Ph*BPL)-assisted biotin identification (PhastID) that meets these criteria and could capture PPIs within minutes of biotin addition. Its fast-labeling kinetics allowed us to map the Rheb dynamic regulatory network upon insulin treatment. Notably, the Rheb PhastID screen identified ATP6AP1, a subunit of the lysosomal V-ATPase complex,^95^ as an activator of Rheb. We demonstrate that ATP6AP1 could bind to Rheb directly and regulate Rheb nucleotide-loading states, independent of its function in maintaining lysosomal pH gradient. Our results support ATP6AP1 as a novel regulator of mTORC1 activation and PhastID as a versatile tool for capturing transient PPIs in live cells.

## Results

### PhastID is a new BioID tool for fast and efficient proximity labeling

**Fig. 1:**
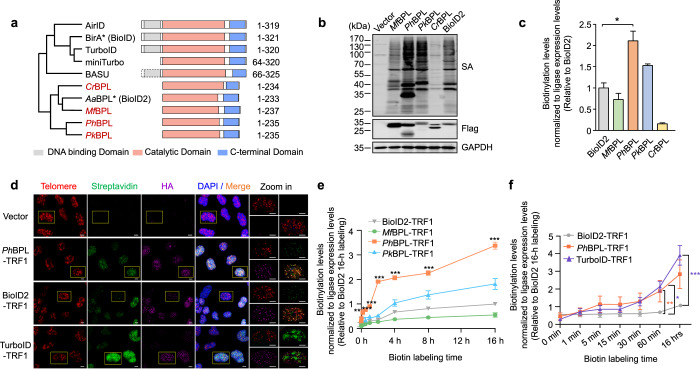
PhastID is fast and efficient for proximity labeling. **a** Domain organization of naturally found (in red) and engineered BPLs examined in this study. BirA*, 35.2 kDa; BASU,^128^ 28.8 kDa; *Cr*BPL, 25.1 kDa; BioID2, 26.6 kDa; *Mf*BPL, 27.4 kDa; *Ph*BPL, 26.5 kDa; *Pk*BPL, 26.3 kDa. TurboID, evolved from EcBirA with multiple mutations. MiniTurbo,^92^ truncated TurboID with mutations. AirID,^129^ de novo designed. **b**, **c** HEK293T cells transiently expressing the indicated HA-Flag-tagged biotin ligases were cultured in the presence of 50 μM biotin for 16 h, and collected for western blot analysis (**b**). SA, Streptavidin Protein DyLight™ 680. GAPDH served as a loading control. Signals from each lane were quantified by ImageJ and presented as mean ± SEM (*n* = 3) (**c**). Total streptavidin signals from each lane were divided by the corresponding Flag signals and then normalized to the BioID2 group. Statistical significance was determined using the two-tailed Student’s *t*-test. *P < 0.05. **d** HeLa cells stably expressing HA-tagged TRF1 fused to different biotin ligases were cultured with 50 μM biotin for 16 h, and then subjected to immunofluorescence analysis with an anti-HA antibody (purple), a telomere probe (TTAGGGTTAGGGTTAGGG) (red), and FITC-conjugated streptavidin (green). Vector alone served as a negative control. DAPI was used to mark the nuclei. Scale bars, 5 μm. Boxed regions are enlarged in zoom-in panels. **e**, **f** Kinetics of different BPLs-TRF1 were compared. The stable cell lines were cultured in dialyzed serum for 3 days, and then added with 50 μM biotin for the indicated times. Cells were harvested for western blot analysis (Supplementary information, Fig. S1c, d). Total streptavidin signals from each lane were divided by the corresponding HA signals and then normalized to the 16-h BioID2 sample as appropriate. Data from all the time points were quantified and plotted (mean ± SEM, *n* = 3). Comparison of new biotin ligases and BioID2 were shown in **e**, and comparison of BioID2-/*Ph*BPL-/Turbo-TRF1 were shown in **f**. Statistical significance was determined using the two-way ANOVA followed by Bonferroni post hoc test. *Ph*BPL-TRF1 showed significantly stronger labeling than BioID2-TRF1 at the indicated time points (**P < 0.01, ***P < 0.001) (**e**). *Ph*BPL-TRF1 showed significantly stronger labeling than BioID2-TRF1 at 60 min (**P < 0.01); TurboID-TRF1 showed significantly stronger labeling than BioID2-TRF1 at 60 min and 16 h (*P < 0.05, ***P < 0.001) (**f**).

Based on homology search with *Escherichia coli* BirA (BioID) and *Aquifex aeolicus* BPL (BioID2) sequences, we selected several prokaryotic biotin ligases and mutated their conserved GRGRXG motifs to GRGGXG for expression and comparison of activity in HeLa cells (Fig. 1a; Supplementary information, Fig. S1a). Most of the new enzymes exhibited promiscuous biotin labeling activities comparable to BioID2, with the group I BPL from the archaebacterium *P. horikoshii* (*Ph*BPL) displaying substantially higher global streptavidin immunoblotting signals (Fig. 1b, c). To further characterize its activity, we fused BioID2, TurboID, or our newly identified BPLs (*Mf/Ph/Pk*) to the N-terminus of human telomeric repeat-binding factor 1 (TRF1) for stable expression in HeLa cells (Supplementary information, Fig. S1b). TRF1 binds to telomeric DNA repeats (TTAGGG) and exhibits characteristic nuclear punctate staining patterns in immunofluorescence assays. TRF1 interacts with multiple telomere-associated factors including TPP1 and forms the six-protein telosome/shelterin complex on telomeres.^96,97^ While TurboID-TRF1 showed diffused nuclear staining, both *Ph*BPL-TRF1 and BioID2-TRF1 signals overlapped extensively with the telomere probe signals (Fig. 1d), indicating that *Ph*BPL tagging did not affect DNA-binding activity or telomere localization of TRF1.

Since fast and efficient labeling was a principal concern to us, we compared labeling kinetics of new BPLs-TRF1, BioID2-TRF1 and TurboID-TRF1 fusion proteins at different time points after biotin addition, starting from as early as 1 min to 16 h. *Ph*BPL exhibited the highest labeling efficiency among the new BPL candidates across the time points examined and biotinylation signal continued to rise (although at a slower rate) after 1 h (Fig. 1e; Supplementary information, Fig. S1c, d), indicating highly efficient biotinylation of endogenous proteins. *Ph*BPL-TRF1 performed better than TurboID-TRF1 within the first 30 min of labeling, while TurboID-TRF1 exhibited the strongest labeling activity with more prolonged labeling (Fig. 1f; Supplementary information, Fig. S1e).

Consistent with previous reports of TurboID cytotoxicity,^94^ stable TurboID-TRF1 expression led to noticeable cell growth inhibition, which likely accounted for its lower expression than *Ph*BPL-TRF1 and BioID2-TRF1 fusion proteins (Supplementary information, Fig. S1f, g). Our results thus far support *Ph*BPL as a promiscuous BPL with low cytotoxicity and high labeling efficiency, making it an ideal tool for probing regulatory interactions that are transient and dynamic. We henceforth named our rapid labeling system *Ph*BPL-assisted identification or PhastID and went on to map the Rheb regulatory interaction network as described below.

### PhastID reveals dynamic Rheb-proximal proteome in response to stimulation

**Fig. 2:**
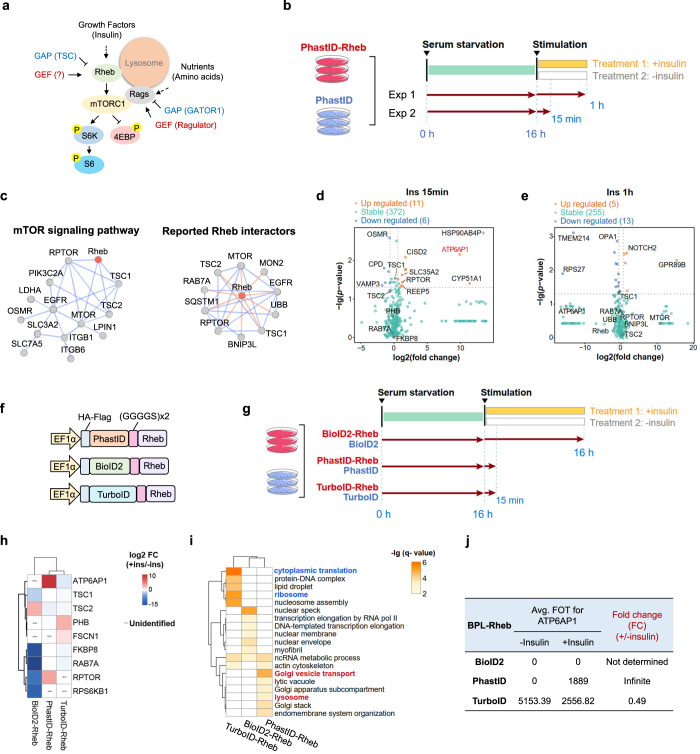
PhastID-mediated proximity labeling reveals dynamic changes in Rheb proteome in response to insulin. **a** Growth factors (e.g., insulin) and nutrients (e.g., amino acids) activate Rheb and Rags that in turn activate mTORC1 to phosphorylate downstream targets such as S6K and 4EBP. **b** HeLa 229 cells stably expressing PhastID-Rheb were serum starved for 16 h and then cultured with 0.9 μM insulin plus 50 μM biotin for 15 min or 1 h, and harvested for streptavidin bead pull-down and LC-MS/MS analysis. PhastID-expressing cells and cells cultured in biotin alone served as controls. Experiments were done in triplicates for each group. **c** Enriched proteins from the 15-min and 1-h treatment groups that have been found in the interacting networks of mTOR signaling pathway or known to interact with Rheb. **d**, **e** Volcano plots of enriched proteins from the 15 min (**d**) or 1 h (**e**) insulin stimulation datasets that were categorized as upregulated (insulin-treated group divided by non-insulin-treated group, fold change > 1.5-fold), downregulated (insulin-treated group divided by non-insulin-treated group, fold change < 0.67-fold), or stable (insulin-treated group divided by non-insulin-treated group, fold change between 0.67- and 1.5-fold). **f** Diagram of the sequence elements in the vectors for BPL-Rheb fusion protein expression as shown. **g** HeLa cells stably expressing PhastID-/TurboID-/BioID2-Rheb were serum starved for 16 h. PhastID-/TurboID-Rheb-expressing cells were cultured with 0.9 μM insulin plus 50 μM biotin for 15 min and BioID2-Rheb-expressing cells for 16 h, and cells were harvested for streptavidin bead pull-down and LC-MS/MS analysis. PhastID-/TurboID-/BioID2-expressing cells cultured in biotin alone served as controls. Experiments were done in triplicates for each group. **h** Heatmap of known regulators of Rheb identified by PhastID-/TurboID-/BioID2-Rheb MS. Fold change was calculated by dividing insulin-stimulated group by non-insulin-treated group from the same biotin ligase. Log_2_ transformed fold change was shown. More than 1.5-fold change was defined as upregulated group. Fold change less than 0.67-fold was defined as downregulated group. Fold change between 0.67- and 1.5-fold was defined as stable group. Known regulators not detected in a biotin ligase are as shown. **i** GO analysis (biological process and cellular component) of upregulated candidates (insulin-treated group divided by non-insulin-treated group, fold change > 1.5-fold) for different BPL-Rheb fusion proteins (PhastID-/TurboID-/BioID2-Rheb) as shown. Top significantly enriched GO terms of each category were integrated as shown. *Q* value was adjusted *P* value using BH adjustment. Terms with *q* value < 0.01 were selected. **j** List of ATP6AP1 relative abundance identified in BioID2-/PhastID-/TurboID-Rheb MS results with or without insulin stimulation, shown as FOT (fraction of total) values. Fold change was calculated by dividing insulin-stimulated group by non-insulin-treated group from the same biotin ligase.

mTORC1 is thought to target to the lysosomal surface through the amino acid-sensitive Rag-Ragulator mechanism where it is in turn activated after binding to lysosomal Rheb^32^ (Fig. 2a). To study the proximity proteins of Rheb, we constructed cell lines stably expressing PhastID-tagged Rheb. Following insulin treatment, phosphorylation of the mTORC1 downstream target S6K appeared to peak after 15 min and began to decrease after 1 h (Supplementary information, Fig. S2a, b). Therefore, we harvested cells at two time points (15 min and 60 min) for pull-down with streptavidin magnetic beads and mass spectrometry (MS) analysis, after addition of insulin and biotin to serum-starved cells (Fig. 2b). The Rheb-proximal proteome appeared distinct from that of the PhastID-only control and showed good congruity between triplicated repeats (Supplementary information, Fig. S2c, d). Among the proteins enriched in the PhastID-Rheb group were several known to be involved in mTORC1/Rheb signaling, including RPTOR,^98^ mTOR,^1–7^ RAB7A,^99^ TSC1,^28^ and TSC2^28^ (Fig. 2c). Proteins that localize to membrane organelles such as lysosomes, endoplasmic reticulum (ER), and the Golgi apparatus were differentially enriched following insulin stimulation (Supplementary information, Fig. S2e, f). These results are in line with the reported subcellular localization of Rheb^65^ and signal the reliability of PhastID at identifying interactions between membrane-bound proteins. Gene ontology (GO) analyses also revealed an enrichment of proteins involved in membrane trafficking and Golgi vesicle transport (Supplementary information, Fig. S2f), which is consistent with the idea that Rheb may also participate in Golgi-related functions.^100^ To better illustrate insulin-induced changes in the Rheb interactome, we divided the identified proteins into three categories: interactions with Rheb upregulated, downregulated, or unchanged (stable) upon insulin treatment (Fig. 2d, e; Supplementary information, Fig. S2e, f). It became clear that some proteins showed different enrichment at different time points following insulin stimulation. For instance, some lysosome-targeted candidates were enriched in the 15-min dataset, but no longer so in the 1-h dataset (Fig. 2d, e). It is possible that some transient interactions with Rheb may be otherwise lost during prolonged stimulation.

To further compare the dynamic labeling efficiency of the present BioID tools, we additionally constructed BioID2- and TurboID-tagged Rheb and individually compared MS data of TurboID- or PhastID-Rheb for 15-min labeling with BioID2-Rheb for 16-h labeling (Fig. 2f, g). Compared to BioID2-Rheb and TurboID-Rheb, PhastID-Rheb was able to correctly identify upregulated RPTOR and ATP6AP1 and downregulated TSC2 as Rheb-proximity proteins showing differential labeling after insulin stimulation (Fig. 2h). Moreover, GO analysis also revealed that lysosome proteins were specifically enriched in PhastID-Rheb proximal proteins after insulin stimulation, in consistence with the model that insulin evokes intracellular vesicle traffic and Rheb activates mTOR at lysosome (Fig. 2i). Besides, long-term BioID2-Rheb labeling failed to identify short-time insulin response components like proteins involved in lysosomal function, e.g., ATP6AP1 (Fig. 2j), and Golgi vesical transport, while TurboID-Rheb was also incapable of achieving dynamic labeling due to high background labeling (e.g., cytoplasmic translation machinery and ribosome) (Fig. 2i). Together, these data further underline the advantages of PhastID over TurboID in identifying dynamic proximal proteomes.

### ATP6AP1 can interact with Rheb and positively regulate mTORC1 signaling

**Fig. 3:**
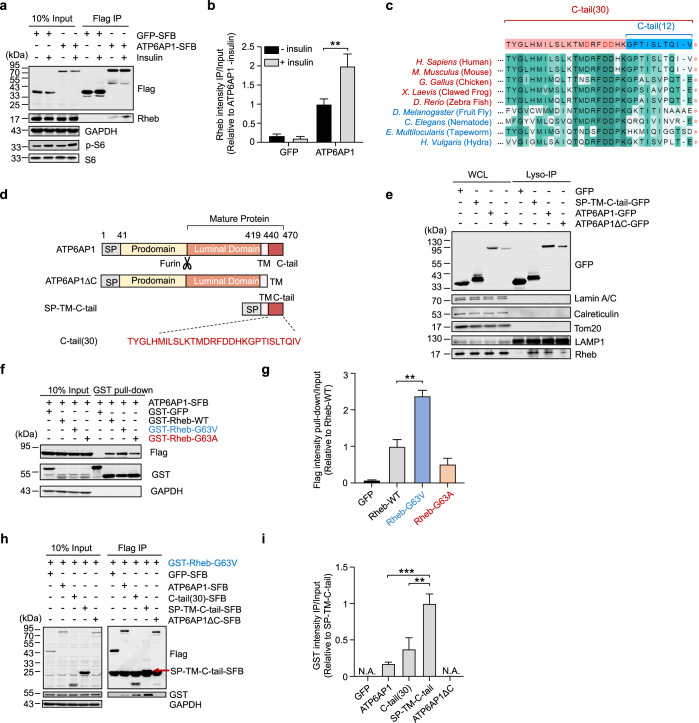
ATP6AP1 binds Rheb. **a**, **b** HEK293T cells stably expressing SFB-tagged ATP6AP1 or GFP were serum starved and then treated with insulin for 15 min. The cells were then collected for immunoprecipitation (IP) with anti-Flag antibody and immunoblotting with the indicated antibodies. Graphed data shown as mean ± SEM (*n* = 3) (**b**). Statistical significance was determined using the two-way ANOVA followed by Dunnett’s multiple comparisons test, compared with ATP6AP1-SFB without insulin group. **P < 0.01. **c** Alignment of ATP6AP1 C-tail sequences across different species. **d** Schematic illustration of WT and mutant ATP6AP1 proteins. SP, lysosomal signaling peptide. TM, transmembrane domain. The site for Furin cleavage in the Luminal domain to produce mature ATP6AP1 is indicated. SP-TM-C-tail, Δaa42–418. C-tail (30), aa 441–470. **e** HEK293T cells stably expressing 3× HA-Flag-tagged TMEM192, with co-expression of Ctrl or *ATP6AP1* shRNAs, were transfected with GFP-tagged full-length ATP6AP1 or its truncation mutants (SP-TM-C-tail or ATP6AP1-ΔC). The cells were subjected to an IP-based lysosome capture process. Whole-cell lysate (WCL) and immunoprecipitated lysosome (Lyso-IP) fractions were immunoblotted to detect the indicated proteins using fluorescent secondary antibodies that removed heavy chains. **f**, **g** HEK293T cells transiently co-expressing ATP6AP1-SFB and the indicated WT or mutant Rheb proteins were collected for GST pull-down and western blotting as indicated. GST-GFP served as a negative control. Graphed data shown as mean ± SEM (*n* = 3) (**g**). Statistical significance was determined using the two-way ANOVA followed by Dunnett’s multiple comparisons test, compared with WT Rheb group. **P < 0.01. **h**, **i** Lysates from HEK293T cells transiently co-expressing GST-tagged Rheb-G63V and SFB-tagged WT or mutant ATP6AP1 were immunoprecipitated with anti-Flag antibody and immunoblotted as shown. Graphed data shown as mean ± SEM (*n* = 3) (**i**). Statistical significance was determined using the two-way ANOVA followed by Dunnett’s multiple comparisons test, compared with SP-TM-C-tail group. **P < 0.01, ***P < 0.001. N.A., not available.

Given the kinetics of mTORC1 activation (Supplementary information, Fig. S2a, b) and that Rheb interaction with its activators is likely stimulated by insulin, the odds are higher that Rheb activators would be found in the upregulated interaction group from the 15-min dataset. One of the highly enriched proteins in PhastID-Rheb experiment following 15-min insulin stimulation was ATP6AP1 (also known as Ac45) (Fig. 2h, j), a lysosomal V-ATPase subunit that acts as a structural hub for Vo complex assembly by connecting multiple Vo subunits and phospholipids in the c-ring.^95,101^ V-ATPases are proton pumps that help maintain the acidification of lysosomal compartments,^102^ and are central players in energy-^103^ and nutrient-sensing functions^80,104^ within cells and implicated in pathophysiological processes such as cancer,^105^ neurodegenerative diseases,^106^ and diabetes.^107,108^ The lysosomal V-ATPase has been shown to form a complex with Ragulator at the lysosome and is necessary for amino acid-mediated mTORC1 activation,^80^ but the role of V-ATPase or its component proteins in regulating the mTORC1 pathway in response to insulin remains largely unknown. We confirmed that both endogenously and exogenously expressed Rheb could co-immunoprecipitate ATP6AP1, and that insulin stimulation led to increased Rheb proteins co-immunoprecipitating ATP6AP1 (Fig. 3a, b; Supplementary information, Fig. S3a, b). As a transmembrane protein, only the C-terminal 30 amino acids (C-tail) of ATP6AP1 are exposed to the cytosol^101^ (Supplementary information, Fig. S3c). This is a region highly conserved from worm to human, including residues DRFDD, Q468 and a TISLT motif (Fig. 3c). When the C-tail was deleted (Fig. 3d), the co-localization of ATP6AP1 with Rheb on lysosomes was significantly diminished according to Lyso-IP and immunostaining assay (Fig. 3e; Supplementary information, Fig. S3d, e). Interestingly, C-tail could not co-immunoprecipitate other small G protein like Ras or Rho, indicating that the interaction between ATP6AP1 C-tail and Rheb is specific (Supplementary information, Fig. S3f).

The consensus DXXG residues in the Rheb switch-II (or G3) region help mediate conformational changes associated with its GTP/GDP state transition^109^ (Supplementary information, Fig. S4a). We therefore generated a series of Rheb mutants with key conserved residues mutated and examined their effects on S6 phosphorylation. When G63 was mutated to Ala or Val, Rheb-G63A but not Rheb-G63V could increase S6 basal phosphorylation (Supplementary information, Fig. S4b), suggesting that the G63A mutation rendered Rheb constitutively active while the G63V mutant remained in the inactive state.^102^ Consistent with this idea, GTPγS beads could pull down substantially more recombinant Rheb-G63A than Rheb-G63V proteins (Supplementary information, Fig. S4c). Interestingly, ATP6AP1 was able to bring down more Rheb-G63V than either wild-type (WT) Rheb or Rheb-G63A (Fig. 3f, g), indicating a preference of ATP6AP1 for the inactive form of Rheb. Indicative of the importance of the C-tail to ATP6AP1–Rheb association, deleting the C-tail almost abolished ATP6AP1 interaction with both Rheb-G63A and Rheb-G63V (Supplementary information, Fig. S4d–f). In fact, the C-tail alone could co-immunoprecipitate Rheb and this interaction or the co-localization with Rheb on lysosome was enhanced when the lysosome-targeting sequence (SP-TM) was added to the C-tail (Fig. 3h, i; Supplementary information, Fig. S3d, e). When a synthetic C-tail peptide was used, more bacterially purified Rheb-G63V proteins could be brought down (Supplementary information, Fig. S4g), implying direct interactions between Rheb and the ATP6AP1 C-tail.

**Fig. 4:**
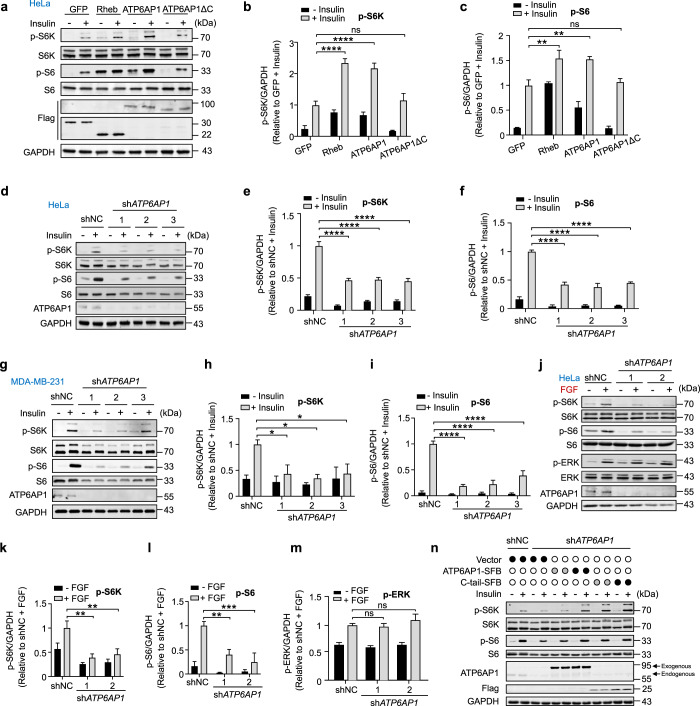
ATP6AP1 regulates mTORC1 signaling. **a**–**c** Serum-starved HeLa cells transiently expressing Flag-tagged Rheb, ATP6AP1 or ATP6AP1ΔC were cultured with 0.9 μM insulin for 15 min, and collected for western blot analysis using the indicated antibodies (**a**). Flag-GFP-expressing cells with insulin treatment served as negative controls. GAPDH was used as a loading control. Intensity values were obtained using ImageJ. p-S6K (**b**) and p-S6 (**c**) signals were normalized to total S6K/S6 as well as GAPDH signals and graphed as mean ± SEM (*n* = 3). Statistical significance was determined using the two-way ANOVA followed by Dunnett’s multiple comparisons test. **P < 0.01, ****P < 0.0001. ns, not significant. **d**–**f** HeLa cells stably expressing *ATP6AP1-*targeting shRNAs were serum starved for 16 h and then cultured with 0.9 μM insulin for 15 min, and cells were collected for western blotting (**d**). shNC, negative control shRNA. GAPDH served as a loading control. Intensity values for p-S6K (**e**) and p-S6 (**f**) were similarly processed to **a**–**c** and graphed as mean ± SEM (*n* = 3). Statistical significance was determined using the two-way ANOVA followed by Sidak’s multiple comparisons test. ****P < 0.0001. shNC with insulin treatment served as a negative control. **g**–**i** MDA-MB-231 cells stably expressing *ATP6AP1-*targeting shRNAs were treated and analyzed as described in **d**–**f**. Statistical significance was determined using the two-way ANOVA followed by Dunnett’s multiple comparisons test. *P < 0.05, ****P < 0.0001. **j**–**m** HeLa cells stably expressing *ATP6AP1-*targeting shRNAs were serum starved for 16 h and then treated with 200 ng/mL FGF plus 50 ng/mL heparin for 15 min, and cells were harvested for western blotting and data analysis as described in **d**–**f**. Statistical significance was determined using the two-way ANOVA followed by Dunnett’s multiple comparisons test. **P < 0.01, ***P < 0.001. ns, not significant. **n** Construct encoding shRNA-resistant SFB-tagged full-length ATP6AP1 or its C-tail alone mutant was transfected at different amount into HeLa cells stably expressing an *ATP6AP1*-targeting shRNA. The cells were serum starved and then cultured with 0.9 μM insulin for 15 min, and harvested for western blot analysis. The depth of the circle color represents different doses. Dark circles represent high doses (4 μg) of the indicated plasmids per well for transfection, while gray circles denote 2 μg of plasmids. The hollow white circles indicate no addition of relevant plasmid.

Even in the absence of insulin, increased phosphorylation of the mTORC1 downstream targets S6K and S6 could be observed in serum-starved HeLa cells overexpressing either Rheb or ATP6AP1, but not ATP6AP1ΔC (Fig. 4a–c). Insulin stimulation led to further increases in S6K and S6 phosphorylation in these cells except for ATP6AP1ΔC-overexpressing cells (Fig. 4a–c). Conversely, although the effect of possibly damaged proton pump on mTORC1 cannot be temporarily ruled out, knockdown of *ATP6AP1* indeed lowered mTORC1 activity as the phosphorylation of S6K and S6 was significantly reduced after insulin stimulation by stable expression of short-hairpin RNA (shRNA) targeting *ATP6AP1* in different cell lines (Fig. 4d–i). Additionally, knockdown of *ATP6AP1* also inhibited fibroblast growth factor (FGF)-induced mTORC1 activation, while FGF-induced ERK activation was not affected (Fig. 4j–m). These results together support the notion that ATP6AP1 could positively regulate growth factor-activated mTORC1 signaling. Moreover, knockdown of *TSC2* could not rescue sh*ATP6AP1*-induced mTORC1 inactivation, suggesting that ATP6AP1 functions downstream of or parallel to TSC2 (Supplementary information, Fig. S4h–j). Overexpressing LAMP1 or LAMP2 alone could not increase S6K and S6 phosphorylation. However, ectopic expression of LAMP1-C-tail or LAMP2-C-tail fusion proteins significantly enhanced the phosphorylation of S6K and S6, indicating that C-tail has the potential to activate mTORC1 pathway via converting Rheb to GTP-bound state even when fused to other lysosomal membrane proteins (Supplementary information, Fig. S4k–m). When the ATP6AP1 C-tail alone was expressed in cells, enhanced mTORC1 activation in response to insulin could be observed dose-dependently (Supplementary information, Fig. S4n). Importantly, expression of shRNA-resistant full-length ATP6AP1 or C-tail alone was enough to restore insulin-induced mTORC1 activation in *ATP6AP1*-knockdown cells also in a dose-dependent manner (Fig. 4n). Collectively, these results not only demonstrate for the first time the interaction between Rheb and ATP6AP1 through the C-tail of ATP6AP1, but also illustrate the importance of ATP6AP1 in Rheb activation in response to growth factor stimulation.

### ATP6AP1 acts as a de facto Rheb GEF through its C-tail

**Fig. 5:**
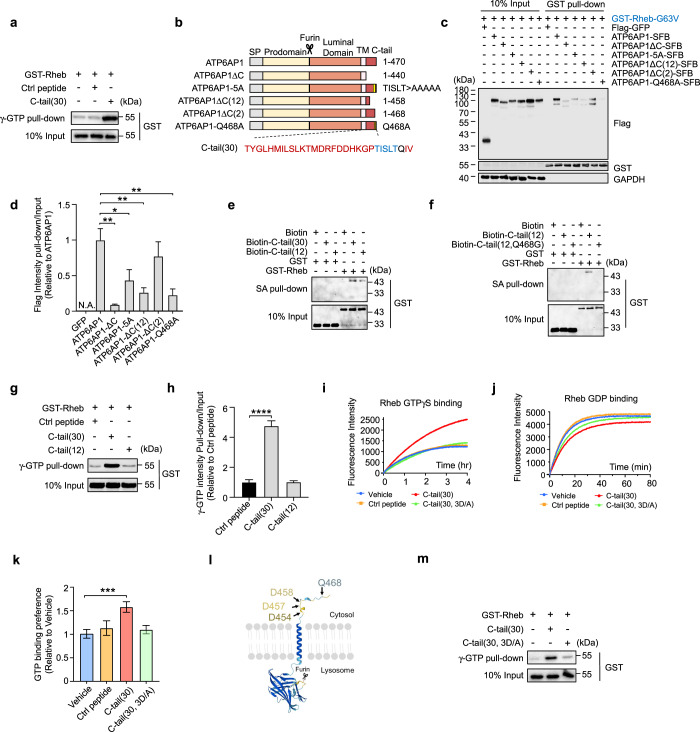
ATP6AP1 functions as a Rheb GEF. **a** Bacterial recombinant GST-tagged WT Rheb proteins were incubated first with a synthetic peptide containing the Rheb C-tail (30) sequence and then pulled down with γ-GTP beads. A synthetic 3× Flag peptide served as control. **b** A series of ATP6AP1 mutants with mutations in the C-tail region were generated. ATP6AP1-5A has the TISLT motif mutated to Ala. **c**, **d** HEK293T cells transiently co-expressing GST-tagged Rheb-G63V and various SFB-tagged ATP6AP1 mutants from **b** were harvested for GST pull-down and immunoblotting. Graphed data shown as mean ± SEM (*n* = 3) (**d**). Statistical significance was determined using the two-way ANOVA followed by Dunnett’s multiple comparisons test, compared with WT ATP6AP1 group. *P < 0.05, **P < 0.01. N.A., not available. **e**, **f** In vitro streptavidin (SA) bead pull-down was performed using mixtures of bacterially purified GST-tagged Rheb proteins and biotinylated synthetic peptides of the ATP6AP1 C-tail (30) and C-tail (12) (**e**), or the C-tail (12) and C-tail (12) with the Q468G mutation (**f**). The precipitates were resolved by SDS-PAGE and immunoblotted as indicated. **g**, **h** Bacterially purified GST-tagged WT Rheb proteins were incubated with synthetic peptides containing the ATP6AP1 C-tail (30) or C-tail (12) sequences for pull-down with γ-GTP beads as done in **a**. A synthetic 3× Flag peptide served as control. Graphed data shown as mean ± SEM (*n* = 3) (**h**). Statistical significance was determined using the two-way ANOVA followed by Dunnett’s multiple comparisons test. ****P < 0.0001. **i**–**k** Recombinant WT Rheb proteins were incubated with BODIPY-FL-GTPγS (**i**) or BODIPY-FL-GDP (**j**) in the presence of WT C-tail (30) or mutant C-tail (30, 3D/A) peptides. Vehicle alone and a Ctrl peptide served as controls. In the analogs, BODIPY-FL fluorescence was quenched by > 90% without G-protein binding. Changes in fluorescence intensity as a result of Rheb binding were monitored in real time and plotted as shown. GTP-binding signals for each sample group were normalized to GDP-binding signals to obtain the values for GTP binding preference (**k**) and presented as mean ± SEM (*n* = 3). Statistical significance was determined using the unpaired two-tailed Student’s *t*-test. ***P < 0.001. **l** The structure of ATP6AP1 as predicted by AlphaFold. D454, D457, D458, and Q468 in the C-tail region are highlighted. **m** Recombinant GST-tagged WT Rheb proteins were incubated with synthetic peptides of WT ATP6AP1 C-tail (30) or the C-tail with 3D/A mutations for pull-down with γ-GTP beads as done in **a**.

The ability of ATP6AP1 to directly bind and activate Rheb makes it a Rheb GEF candidate despite a lack of canonical GEF domains. To further explore this possibility, we used GTPγS beads to pull down bacterially purified GST-Rheb proteins in the presence or absence of a synthetic ATP6AP1 C-tail peptide. The presence of the C-tail peptide significantly increased the amount of Rheb proteins that associated with GTPγS (Fig. 5a), suggesting that the ATP6AP1 C-tail was able to facilitate Rheb transition to the GTP-bound form. Complete or partial deletion of the ATP6AP1 C-tail as well as mutations of the highly conserved Q468 or TISLT motif (Fig. 5b; Supplementary information, Fig. S5a) in this region either abrogated or drastically reduced its interaction with Rheb (Fig. 5c, d), adding more support to the idea that ATP6AP1–Rheb interaction through the C-tail promotes Rheb transition to the active form and that the conserved motif and residues in the C-tail are critical to ATP6AP1–Rheb interaction. In fact, just the last 12 residues (C12) of ATP6AP1 alone could still bind Rheb, and the Q468G mutation blocked this interaction (Fig. 5e, f). However, the synthetic C12 peptide failed to promote GTP binding of Rheb (Fig. 5g, h), signaling a division of labor within the ATP6AP1 C-tail where the terminal 12 amino acids mediate Rheb interaction while the upstream sequences facilitate Rheb binding to GTP.

**Fig. 6:**
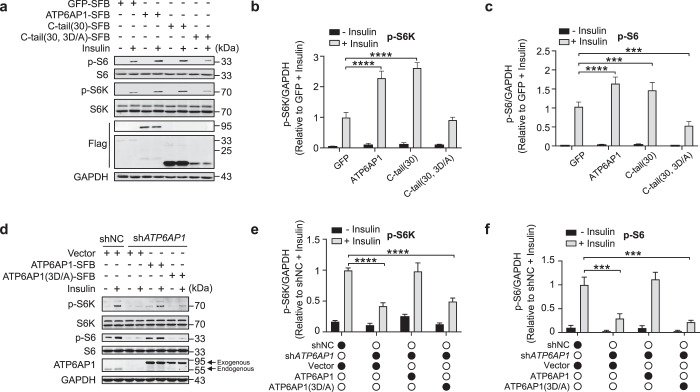
ATP6AP1 regulates mTORC1 through Rheb. **a**–**c** HeLa cells transiently expressing the indicated SFB-tagged WT ATP6AP1 or C-tail mutants were serum starved and then treated with 0.9 μM insulin for 15 min, and harvested for western blot analysis (**a**). Intensity values were obtained using ImageJ. p-S6K (**b**) and p-S6 (**c**) signals were normalized to total S6K/S6 as well as GAPDH signals and graphed as mean ± SEM (*n* = 3). Statistical significance was determined using the two-way ANOVA followed by Dunnett’s multiple comparisons test. ***P < 0.001, ****P < 0.0001. GAPDH served as a loading control. GFP with insulin treatment group served as a negative control. **d**–**f** Stable *ATP6AP1*-knockdown HeLa cells that also expressed shRNA-resistant WT or mutant ATP6AP1 were examined as in **a**. Intensity values for p-S6K (**e**) and p-S6 (**f**) were similarly processed to **a**–**c** and graphed as mean ± SEM (*n* = 3). Statistical significance was determined using the two-way ANOVA followed by Tukey’s multiple comparisons test. ***P < 0.001, ****P < 0.0001. shNC with insulin treatment group served as a negative control.

To investigate Rheb GTP loading in more detail, we turned to BODIPY-FL-conjugated GTP/GDP analogs, whose association with G proteins enables BODIPY-FL fluorescence recovery and real-time monitoring of GTP/GDP binding^110^ (Supplementary information, Fig. S5b). Consistent with our data above, in the presence of the ATP6AP1 C-tail peptide, dramatic increases in fluorescence intensities could be detected in binding reactions with the GTP analog compared to vehicle or control peptides, indicating enhanced Rheb GTP binding (Fig. 5i–k). It has been shown that GEFs often insert acidic residues into the Mg^2+^- and nucleotide-binding loop of G proteins to facilitate guanine nucleotide release and disrupt the binding of phosphate and metal ions with G proteins.^111,112^ The ATP6AP1 C-tail contains three highly conserved aspartate residues (Fig. 5l). Given that consecutive aspartate residues can bind the Mg^2+^ cation,^113^ we generated mutants with these residues replaced by alanine. The ATP6AP1 C-tail tri-aspartate mutant (3D/A) still retained the ability to bind Rheb (Supplementary information, Fig. S5c) but failed to promote Rheb GTP binding that would allow sufficient BODIPY-FL fluorescence recovery (Fig. 5i, k) and pull-down of Rheb by γ-GTP beads (Fig. 5m). In binding reactions with the GDP analog, a slight decrease in fluorescence intensity could be observed in the presence of the WT C-tail peptide but not the tri-aspartate mutant (Fig. 5j), suggesting that C-tail binding may help deter Rheb association with GDP as well. These findings point to ATP6AP1 as a de facto Rheb GEF and underscore the importance of the C-tail and the conserved tri-aspartate residues to its GEF activity. Indeed, the C-tail 3D/A mutant could no longer facilitate mTORC1 signaling (Fig. 6a–c). When shRNA-resistant full-length ATP6AP1 that harbored the 3D/A mutations was expressed in *ATP6AP1*-knockdown cells, it also failed to rescue phosphorylation of S6K, S6 or 4EBP, indicator for mTORC1 activation, upon insulin stimulation (Fig. 6d–f; Supplementary information, Fig. S5d, e). As expected, reconstitution with the 3D/A mutations also led to AMPK activation (Supplementary information, Fig. S5f, g), but did not affect the recruitment of mTORC1 to the lysosome (Supplementary information, Fig. S5h, i).

We found that *Rheb* knockdown enhanced the effect of sh*ATP6AP1* on mTORC1 signaling activity, as all of the phosphorylation levels of S6K, S6 and 4EBP further decreased (Supplementary information, Fig. S5j–m), further demonstrating the function of ATP6AP1 as a GEF specifically towards Rheb. When the constitutively active form of Rheb (CA-Rheb) or RagB (CA-RagB) was overexpressed in *ATP6AP1*-knockdown cells, CA-Rheb could rescue mTORC1 activation that was inhibited by the loss of *ATP6AP1* (Supplementary information, Fig. S5n–o). These data strongly suggest that ATP6AP1-induced mTORC1 activation occurs through Rheb. Collectively, our results thus far provide evidence that the ATP6AP1 C-tail mediates the Rheb GEF function, which relies on the last 12 amino acids (C12) for Rheb interaction and the tri-aspartate motif (D454/D457/D458) for promoting nucleotide exchange (Supplementary information, Fig. S5p).

Next, we used AlphaFold-Multimer^114^ to model the possible three-dimensional structure of Rheb/ATP6AP1 C-tail (30) peptide complex. Interestingly, one reasonable model is consistent with our experimental results (Supplementary information, Fig. S6a–d). Notably, the interaction between C-tail Q28 (ATP6AP1 Q468) and the Rheb E40 residue in the switch I region goes well with our results that the C-tail Q28A mutation blocked the Rheb–ATP6AP1 interaction (Fig. 5c, d; Supplementary information, Fig. S6e). Moreover, C-tail I24 and L26 (ATP6AP1 I464 and L466) exhibit hydrophobic interactions with Rheb I24, V27 and F43 residues, respectively (Supplementary information, Fig. S6f), consistent with the results that the mutation of ATP6AP1 TISLT motif into five alanines (5A) significantly reduced the ATP6AP1 interaction with Rheb (Fig. 5c, d). Importantly, C-tail D17 residue drilling into the switch I loop binds Mg^2+^, thereby reducing the ability of Mg^2+^ to stabilize β-phosphate of GDP and making GDP more easily dissociated (Supplementary information, Fig. S6g–k), thus functioning as GEF.

### ATP6AP1 mutants inhibit cancer cell growth and migration

ATP6AP1 appears to be upregulated in multiple cancer types, especially breast invasive carcinoma, kidney chromophobe, and skin cutaneous melanoma (Supplementary information, Fig. S7a, b). Higher ATP6AP1 expression is also correlated with more advanced cancer stage and poorer prognosis in breast cancer patients (Supplementary information, Fig. S7c, d). We found that protein levels of ATP6AP1 were elevated in multiple breast cancer cell lines, such as MDA-MB-231, MDA-MB-436, and MCF7, compared with the non-tumorigenic MCF10A cell line (Supplementary information, Fig. S7e). In both ER^+^ and triple-negative human breast adenocarcinoma cell lines (MCF7 and MDA-MB-231), stable ATP6AP1 depletion led to decreased anchorage-independent growth (Supplementary information, Fig. S7f, g), and reduced migration in both wound healing (Supplementary information, Fig. S7h, i) and transwell migration/invasion assays (Supplementary information, Fig. S7j, k). Consistent with ATP6AP1 being a key subunit of V-ATPase,^101^ *ATP6AP1* knockdown also disrupted lysosome acidification as evidenced by increased lysosome pH in these cells (Supplementary information, Fig. S7l, m).

**Fig. 7:**
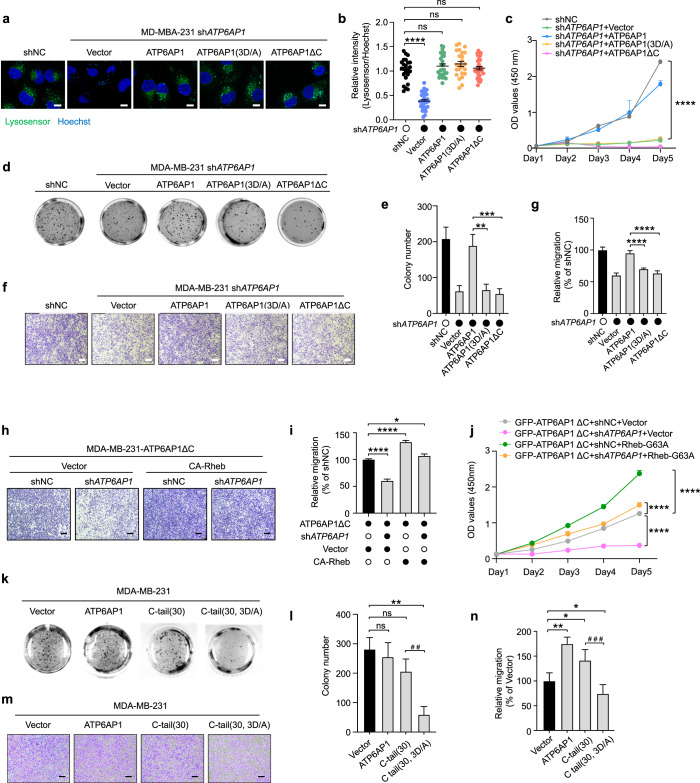
Targeting ATP6AP1 inhibits cancer cell growth and migration. **a,** **b** Stable *ATP6AP1*-knockdown MD-MBA-231 cells expressing full-length or mutant ATP6AP1 were labeled with the green fluorescent LysoSensor Dye to monitor lysosomal pH (**a**). The LysoSensor dye is nearly non-fluorescent unless residing inside acidic organelles such as lysosomes. Hoechst 33342 (C1022, Beyotime) (blue) was used to stain the nuclei. Scale bars, 5 μm. Relative fluorescence intensity was obtained by dividing green fluorescence signals by Hoechst staining signals and plotted as mean ± SEM by GraphPad Prism (**b**). shNC, non-targeting shRNA. Statistical significance was determined using the two-way ANOVA followed by Dunnett’s multiple comparisons test. ****P < 0.0001. ns, not significant. **c** The growth of cells from **a** was examined by the CCK8 assay. Cell number was determined at the indicated time points and plotted by GraphPad Prism as mean ± SEM (*n* = 3). Statistical significance was determined using the two-way ANOVA followed by Dunnett’s multiple comparisons test. ****P < 0.0001. **d**, **e** Cells from **a** were used for colony formation assays in soft agar. Cell colonies were visualized (**d**) and quantified (**e**) as mean ± SEM (*n* = 3). Vector alone and shNC served as negative controls. Statistical significance was determined using the two-way ANOVA followed by Dunnett’s multiple comparisons test. **P < 0.01, ***P < 0.001. **f**, **g** Cells from **a** were examined in transwell migration assays (**f**). Scale bars, 100 μm. Cell migration was quantitated and presented as mean ± SEM (*n* = 3) (**g**). Vector alone and shNC served as negative controls. Statistical significance was determined using the two-way ANOVA followed by Dunnett’s multiple comparisons test. ****P < 0.0001. **h**–**j** MDA-MB-231 cells stably expressing the *ATP6AP1*-targeting shRNA along with GFP-tagged ATP6AP1 with C-terminal deletion and HA-Flag-tagged vector or CA-Rheb (constitutively active form of Rheb) were serum starved for 16 h and then stimulated with 0.9 μM insulin for 15 min. Cells were examined by transwell migration assays (**h**) and quantitated (**i**) as mean ± SEM (*n* = 3). Scale bars, 100 μm. shNC plus vector served as a negative control. Statistical significance was determined using the two-way ANOVA followed by Dunnett’s multiple comparisons test. *P < 0.05, ****P < 0.0001. The growth of cells from **h** was examined by the CCK8 assay (**j**). Cell number was determined at the indicated time points and plotted by GraphPad Prism as mean ± SEM (*n* = 3). Statistical significance was determined using the two-way ANOVA followed by Dunnett’s multiple comparisons test. ****P < 0.0001. **k**, **l** MD-MBA-231 cells stably expressing full-length ATP6AP1, WT C-tail (C-tail (30)) or mutant C-tail (C-tail (30, 3D/A)) were examined by colony formation assays (**k**). Colony numbers were counted and graphed as mean ± SEM (*n* = 3) (**l**). Vector alone served as negative control. Statistical significance was determined using the two-tailed Student’s *t*-test. **P < 0.01, ^##^*P* < 0.01. ns, not significant. **m**, **n** Cells from **k** were examined by transwell migration assay (**m**). Scale bars, 100 μm. Cell migration was quantitated and presented as mean ± SEM (*n* = 3) (**n**). Vector alone served as negative control. Statistical significance was determined using the two-tailed Student’s *t*-test. *P < 0.05, **P < 0.01, ^*###*^*P* < 0.001.

To rule out the possibility that lowered mTORC1 activity was due to the damaged proton pump caused by *ATP6AP1* knockdown in this study (Fig. 4d–i), and also to probe whether the GEF activity and proton pump function of ATP6AP1 are linked, we expressed shRNA-resistant ATP6AP1 C-tail mutants in the *ATP6AP1*-knockdown cells. As expected, expression of WT ATP6AP1 was able to restore proper lysosome acidification (Fig. 7a, b) as well as mTORC1 signaling (Supplementary information, Fig. S8a–c). Expression of the ΔC-tail and tri-aspartate mutants could restore low lysosomal pH as well (Fig. 7a, b), indicating that the C-tail is dispensable for V-ATPase-mediated lysosomal pH gradient maintenance. On the other hand, mTORC1 signaling remained dysfunctional in *ATP6AP1*-knockdown cells expressing the ΔC or 3D/A mutant (Supplementary information, Fig. S8a–c), suggesting that GEF and proton pump functions are modular activities mediated by separate domains of ATP6AP1 and that the C-tail is essential to ATP6AP1-dependent Rheb activation. Consistent with these data, expression of WT ATP6AP1 but not the two mutants in *ATP6AP1*-knockdown cells could restore proliferation (Fig. 7c), anchorage-independent growth (Fig. 7d, e), and migration (Fig. 7f, g; Supplementary information, Fig. S8d, e), providing further evidence that the Rheb GEF activity of ATP6AP1 is critical for cancer cell proliferation and migration.

To determine whether the effect of ATP6AP1 loss of function on mTORC1 activation is truly attributed to the lack of Rheb activation, we expressed constitutively active form of Rheb (CA-ooRheb) in *ATP6AP1*-knockdown cells carrying the ATP6AP1ΔC mutant (Supplementary information, Fig. S8f). Expression of CA-Rheb in these cells could restore migration in wound healing (Supplementary information, Fig. S8g, h), transwell migration/invasion (Fig. 7h, i), and proliferation assays (Fig. 7j), suggesting that the effects of ATP6AP1 deficiency on cancer cell growth and migration were indeed dependent on Rheb activity. Notably, overexpressing the C-tail tri-aspartate mutant peptide (C-tail (30, 3D/A)) alone was able to inhibit cancer cell anchorage-independent growth (Fig. 7k, l) and migration (Fig. 7m, n; Supplementary information, Fig. S8i, j). The GEF activity of ATP6AP1 therefore may represent an alternative avenue for modulating mTORC1 signaling and a potential therapeutic target for blocking mTORC1 signaling and inhibiting tumorigenesis. Taken together, our findings combined support a model in which the lysosomal V-ATPase protein ATP6AP1 acts as a Rheb GEF to regulate mTORC1 activation.

## Discussion

In this study, we developed the *Ph*BPL-based PhastID tool for rapid, efficient, and robust labeling of endogenous proteins in live cells and for investigating dynamic and regulatory PPIs. We followed the BioID protocol described in Branson et al.^92^ which is more in favor of soluble proteins. However, we still found several membrane proteins enriched in our captured list. Future usage of higher detergent concentrations may help improve application of PhastID in membrane protein identification. Similar to BioID2, PhastID does not have any DNA-binding domains, which should help reduce non-specific binding.^115,116^ Additionally, *P. horikoshii* is thermophilic, and its BPL therefore may be more stable and better expressed in cells. Indeed, compared to TurboID, fewer protein folding-related targets were labeled by PhastID. In addition, this hyperthermostability makes PhastID a good candidate for usage under more extreme environmental conditions. We envision multiple improvements in PhastID that should further expand its utility. For example, *Ph*BPL may be truncated or modified to generate more compact versions of PhastID that would minimize mis-localization and allow more efficient targeting of fusion proteins. Expanding its labeling capability to nucleic acids as in the case with APEX2 is another possibility.^93^ Computational studies such as direct evolution of PhastID may achieve even faster and more efficient labeling and thus further broaden the scope of its application.

Proteins including TCTP^87^ and PAM^117^ have been thought to directly activate Rheb, but it remains unclear whether they are true GEFs of Rheb. For instance, there is evidence that TCTP cannot facilitate the transition of GDP-bound Rheb to GTP-bound Rheb.^88^ PAM is mainly regulated by sphingosine-1-phosphate and interacts with TSC2,^117^ but does not appear to directly interact with Rheb. It is also worth noting that these two proteins were not enriched in either the 15-min or 1-h datasets. PhastID identified the V-ATPase subunit ATP6AP1 as a Rheb-interacting protein, whose interaction with Rheb was upregulated shortly after growth factor stimulation. Our data show that ATP6AP1 functions as a de facto GEF for Rheb. Given the membrane localization of ATP6AP1, this conclusion is also supported by experimental evidence suggesting that Rheb GEF(s) is most likely localized at or near the membrane.^84^ Our findings may help explain why Rheb can remain highly activated (4–8-fold higher than other Ras family proteins) even during serum starvation.^118^ We postulate that such high basal activities may be sustainable due to the close proximity of Rheb and ATP6AP1 on the lysosomal membrane.

Rheb is localized in multiple membrane organelles including the ER, Golgi apparatus, and lysosomes. Our data suggest that the lysosome is where Rheb activation occurs in response to growth factor stimulation. ATP6AP1 lacks canonical GEF domains and instead employs the tri-aspartate motif in its C-tail to control Rheb activity. The ATP6AP1 C-terminal sequence is highly conserved, hinting at evolutionary conservation of its regulatory function on Rheb or Rheb-like proteins. While canonical GEFs usually decrease G-protein nucleotide binding affinity without favoring the reloading of GTP or GDP,^78^ ATP6AP1 appeared to enhance Rheb binding with GTP while deterring its association with GDP, thus promoting GDP-GTP exchange. A recent study showed that ATP6AP1 was targeted by PEN2 for the activation of lysosomal AMPK with low doses of metformin,^104^ and that *ATP6AP1* knockout led to AMPK activation. PEN2 can bind ATP6AP1 via its transmembrane domain and is thus docked on the V-ATPase.^104^ Taken together with our finding that ATP6AP1 inhibition could block growth factor-induced mTORC1 signaling, these data place ATP6AP1 at the center of two antagonistic signaling pathways, mTORC1 and AMPK. Given the important role of V-ATPase in cellular and pathophysiological processes, more work is needed to elucidate the crosstalk on lysosomes between these two signaling cascades and how that translates into cellular responses. Abnormally active mTORC1 signaling cascades may enable the proliferation of cancer cells even in nutrient-poor environments. Although cell migration is considered to be regulated by mTORC2,^32^ our results together with some other reports also suggest a possible role of mTORC1 in cancer or immune cell migration.^119–124^ Therefore, mTOR inhibitors (e.g., Everolimus and Temsirolimus) are being actively investigated as cancer therapeutics.^125^ Our identification of ATP6AP1 as a regulator and de facto GEF of Rheb may also have important clinical implications. Whether functional peptides and/or small molecules derived from ATP6AP1 can serve as specific inhibitors of mTORC1 pathways warrants further investigation that may yield therapeutic alternatives for cancer and disease treatment.

## Materials and methods

### Vectors

cDNAs encoding WT and mutant Rheb and ATP6AP1 were cloned into bacterial expression vectors for N-terminal GST or His tagging (pDEST27 and pET28a, Invitrogen), and eukaryotic expression vectors for N-terminal tagging with Flag-HA (pLenti-FLHA) or C-terminal tagging with SFB (S-tag, Flag and streptavidin-binding protein) (pLentiSFB).

cDNAs encoding various biotin ligases were cloned into the pcDNA3.1 vector for C-terminal Flag-HA tagging. *TRF1* cDNA sequences were fused to cDNAs encoding various biotin ligases through a G4S linker and cloned into the pLenti vector for N-terminal HA-Flag tagging. Biotin ligases fused to a nuclear localization sequence were used as negative controls. All the constructs in this study have been sequence verified by Sanger sequencing. The pLKO.1 vector (#32682, Addgene) was used to clone the following shRNA sequences.

shNC: CCTAAGGTTAAGTCGCCCTCG;

sh*ATP6AP1*-1: GCATTGAGGATTTCACAGCAT;

sh*ATP6AP1*-2: TGCAGCTCTCTACCTACTTAG;

sh*ATP6AP1*-3: ACAGTGACATTCAAGTTCATT;

sh*ATP6AP1*-3′-UTR: TAAGAAGTACACGGGTTTATT.

### Cell lines and cell treatment

Low-passage HeLa, HeLa 229, MCF7, MDA-MB-231, MDA-MB-436, SUM149, MCF10A and HEK293T cells were purchased from the American Type Culture Collection and cultured in Dulbecco’s modified Eagle’s medium (DMEM) high glucose (Gibco) supplemented with 10% fetal bovine serum (FBS) (Excel Biosciences) at 37 °C with 5% CO_2_. For transient expression, ~3 × 10^5^ cells/well were seeded in 6-well plates the day before transfection. All transfections except HeLa cell transfection were carried out using PEI (24765-1, Polysciences). LipoFectMax^TM^3000 (FP139M, ABP Biosciences) was used for transfection of HeLa cells. Stable cell lines were generated through infection with lentiviruses encoding the desired proteins followed by antibiotic selection as appropriate.

For serum starvation, cells were cultured in DMEM medium without FBS supplementation for 16 h. For growth factor stimulation, serum-deprived cells were cultured as needed, with 0.9 µM insulin (Sigma-Aldrich, Louis, MO, USA) or 12.9 nM FGF (200 ng/mL, #F5542, Sigma) and 50 ng/mL heparin (#9041-08-1, Aladdin), for 15 min. To examine cell proliferation, cells were seeded in 12-well plates at ~10% confluence (~5 × 10^4^ cells/well). Cell number was determined at different time points by counting chamber analysis according to the manufacturer’s instructions.

For biotin labeling, 50 μM biotin (#B4639, Sigma) was added to the culture medium 24 h after transfection for transient expression cells. For large-scale affinity purification using stable expression cell lines, cells were seeded in 15-cm dishes at ~80% confluence (~2 × 10^7^ cells) and cultured with 50 μM biotin for the desired length of time.

### Liquid chromatography (LC)-MS

In MS analysis, we used ~2 × 10^7^ cells per replicate for labeling and pull-down with streptavidin magnetic beads (Pierce, 88816) in triplicate. In the BPL-TRF1 study, following lysis with RIPA buffer, only the nuclear fraction was used for subsequent pull-down with streptavidin beads. In the BPL-Rheb study, whole cell lysates were used for streptavidin bead pull-down following lysis with RIPA buffer immediately after 15-min labeling. Sample preparation for LC-MS analysis was performed essentially as previously described with minor modifications.^115^ Briefly, protein samples were allowed to migrate into a NuPAGE 4%–12% Bis-Tris Protein Gel (Invitrogen, NP0335BOX). Each sample lane was then excised for trypsin digestion overnight. For PhastID-TRF1 MS samples, the extracted peptides were loaded onto an Easy-nLC liquid chromatograph coupled LTQ-Orbitrap Elite mass spectrometer (Thermo Fisher Scientific). Alternatively, PhastID-Rheb MS samples were also analyzed with the Wininnovate Bio company coupled Triple TOF 6600 LC-MS (AB SCIEX). Peptides were eluted and separated with a C18 pre-column (Acclaim PepMap^TM^, 75 μm × 2 cm, nanoViper, 3 μm, 100 Å, Thermo Fisher Scientific) followed by separation on a C18 analytical column (Acclaim PepMap^TM^, 75 μm × 15 cm, nanoViper, 2 μm, 100 Å; Thermo Fisher Scientific) with the gradient conditions: 5%–25% buffer B (0.1% formic acid in 80% acetonitrile and 20% water) for 30 min, 25%–40% buffer B for 15 min, 40%–98% buffer B for 10 min, and 98% buffer B for 5 min, with a constant flow rate of 300 nL/min (buffer A: 0.1% formic acid in ultrapure water). Survey scans of peptide precursors were performed from 350 *m/z* to 1500 *m/z* at 60,000 FWHM resolution (200 *m/z*) in the positive ion mode. For the data-dependent mode, cycle time was set to 3 s. Higher energy C-trap dissociation fragmentation was applied with 30% collision energy and the resulting fragments were detected with the resolution of 15,000. Dynamic exclusion was set to 60 s with a 10-ppm mass tolerance around the precursor and its isotopes. All MS data were collected using the information-dependent analysis mode and deposited in the ProteomeXchange Consortium (http://proteomecentral.proteomexchange.org, Project ID: IPX0005111000, ProteomeXchange ID: PXD037078).

### Recombinant protein purification and in vitro binding assays

GST- or His-tagged WT and mutant Rheb proteins were purified from the *E. coli* BL21 (DE3) strain as previously described.^126^ *E. coli* cells were induced with 0.5 mM IPTG at 25 °C for 16 h, and then cells were lysed and sonicated. The recombinant proteins were pulled down using Glutathione Sepharose 4B (Cytiva, GE17-0756-01) or Ni Sepharose^TM^ 6 Fast Flow (GE Healthcare, 17-5318-01) and eluted using GST or Ni-NTA agarose columns (Beyotime Biotechnology).

For direct binding assays, a 1:5 molar ratio mixture of recombinant proteins (1 µg) and biotinylated C-tail peptides (Genscript ProBio) in 300 µL NETN buffer (40 mM Tris-HCl, 100 mM NaCl, 0.5% NP-40, 1 mM EDTA, 10% glycerol, 1% protease inhibitor cocktail (Bimake, B14001)) were incubated with 15 µL of streptavidin beads for 2 h at 4 °C with gentle rotation. The beads were then washed three times with NETN buffer, once with 1× TBST, and once more with 1× TBS. The bound proteins were eluted in 1× loading buffer (#FD006, Fude Biological Technology) by incubation at 95 °C for 10 min.

For GTP/GDP binding assays, a 1:5 molar ratio mixture of recombinant proteins (1 µg) and biotinylated C-tail peptides (Genscript ProBio) in 100 µL lysis buffer G (1× PBS, pH 7.4, 1% Triton X-100, 1× phosphatase inhibitors (P0044 and P5726, Sigma)) were incubated with 15 µL of γ-(6-aminohexyl)-GTP sepharose suspension (Jena Bioscience) for 1 h at 4 °C, and 2.5 mM GTPγS (20-176, Sigma-Aldrich) and 0.4 mM GDP (20-177, Sigma-Aldrich) were added for another hour of incubation at 4 °C. GTP-bound proteins were then eluted at 70 °C for 10 min in 20 µL of 1× loading buffer (#FD006, Fude Biological Technology) for further analysis.

For real-time GTP/GDP binding assays, recombinant His-tagged Rheb proteins (4.58 μM) were mixed with WT or mutant ATP6AP1 C-tail peptides (22.9 μM, Genscript), and 20 μL of 3 μM BODIPY-FL-GDP (G22183, Invitrogen) or BODIPY-FL-GTPγS (G22360, Invitrogen), plus 10 μL of 12 mM MgCl_2_ and 70 μL of binding buffer (10 mM HEPES, 1 mM EDTA, 10 mM MgCl_2_). Vehicle and a control peptide (22.9 μM, Sigma) were used as controls. Changes in fluorescence intensity were detected at 488 nm using the Multifunctional Microplate Reader (Biotek Synergy HTX).

### Lyso-IP

5 × 10^6^ TMEM192-3× HA-Flag HEK293T cells were seeded in 10-cm dishes. Cells were collected after two days of transfection with control or *ATP6AP1* WT/mutants. Cells were scraped in 1 mL ice-cold PBS containing protease and phosphatase inhibitors, centrifuged at 1000× *g* for 2 min at 4 °C, resuspended in 950 μL PBS and gently homogenized with 20 strokes. Then, 25 μL cell suspension was transferred to a new tube and lysed to generate whole-cell lysate samples. Intact cells were pelleted at 1000× *g* for 2 min, and the supernatant was incubated with 50 μL of pre-washed anti-Flag beads for 2 h with gentle rotation. Immunoprecipitated lysosomes were washed three times with PBS containing protease and phosphatase inhibitors and subsequently lysed.

### Colony formation assays

Cells were seeded in 24-well plates at ~1 × 10^5^ cells/well on 0.5 mL of bottom-layer agarose medium (1:1 mixture of 1.2% low-melting point agarose and pre-warmed 2× DMEM supplemented with 20% FBS) and incubated at room temperature for 60 min. For top-layer agarose medium, cells resuspended in culture medium were mixed with 0.8% agar at a 1:1 ratio and then added to the bottom medium for incubation at room temperature for at least 30 min. The cells were then cultured at 37 °C with 5% CO_2_ for two weeks with addition of 200 µL culture medium twice per week to prevent drying. Colonies were stained with 100 μL of 5 mg/mL MTT (3-(4,5-dimethylthiazol-2-yl)-2,5-diphenyltetrazolium bromide) for 1 h at 37 °C and imaged by NIS-Elements Viewer.

### Transwell migration and invasion assays

The assay was performed as previously described.^127^ Briefly, ~2 × 10^4^ cells/well were seeded onto transwell membrane filter inserts (#3450, Corning) in 24-well plates. After 24 h, the transwell inserts were washed twice with 1× PBS, fixed with 4% paraformaldehyde, and stained with crystal violet for 15 min. Cell migration was visualized and imaged using a phase contrast microscope (Nikon EclipseTs2-FL).

### Wound healing assays

Cells were seeded in complete media in a 12-well plate so that they would reach 100% confluence the next day (~3 × 10^5^ cells/well). A 100-μL pipette tip was used to scratch the cell monolayer and the linear wound was imaged. The cells were then cultured in DMEM plus 2% FBS and maintained at 37 °C with 5% CO_2_ for 12 h for imaging and analysis.

### Structure modeling

The three-dimensional structure of Rheb/ATP6AP1 C-tail (30) peptide complex was predicted by AlphaFold-Multimer using full-length Rheb sequence (NP_005605.1) and C-tail sequence (TYGLHMILSLKTMDRFDDHKGPTISLTQIV). Molecular images were generated by UCSF ChimeraX (v1.2.5). The predicted Rheb/ATP6AP1 C-tail (30) complex was further superimposed onto Rheb-GDP structure (PDB: 1xtq) by ChimeraX (v1.2.5) Matchmaker. The predicted structure was provided in cif format as Supplementary information, Data S2.

### Quantification and statistical analysis

All quantitative experiments were performed with three independent biological repeats unless otherwise indicated. The results were analyzed and graphed using the GraphPad Prism 9 software. All data are presented as mean ± SEM. Unpaired two-tailed Student’s *t*-test was used to compare the means of two groups and calculate *P* values.

Antibodies and other reagents, and other methods are described in Supplementary information, Data S1.

## Supplementary information

Supplementary information, Fig. S1

Supplementary information, Fig. S2

Supplementary information, Fig. S3

Supplementary information, Fig. S4

Supplementary information, Fig. S5

Supplementary information, Fig. S6

Supplementary information, Fig. S7

Supplementary information, Fig. S8

Supplementary information, Data S1

Supplementary information, Data S2
