# Author Correction: The rapid proximity labeling system PhastID identifies ATP6AP1 as an unconventional GEF for Rheb

**DOI:** 10.1038/s41422-025-01088-6

**Published:** 2025-02-28

**Authors:** Ran Feng, Feng Liu, Ruofei Li, Zhifen Zhou, Zhuoheng Lin, Song Lin, Shengcheng Deng, Yingying Li, Baoting Nong, Ying Xia, Zhiyi Li, Xiaoqin Zhong, Shuhan Yang, Gang Wan, Wenbin Ma, Su Wu, Zhou Songyang

**Affiliations:** 1 MOE Key Laboratory of Gene Function and Regulation, State Key Laboratory of Biocontrol, Guangzhou Key Laboratory of Healthy Aging Research, School of Life Sciences, Institute of Healthy Aging Research, Sun Yat-sen University, Guangzhou, Guangdong China; 2 Sun Yat-sen Memorial Hospital, Sun Yat-sen University, Guangzhou, Guangdong China

**Keywords:** Insulin signalling, TOR signalling, Proteomic analysis

Correction to: *Cell Research* 10.1038/s41422-024-00938-z, published online 06 March 2024

It has come to our attention that in our paper published in *Cell Research* in 2024, the picture of Supplementary information, Fig. S8i (vector group, 0 h) was mistakenly reused from that of Supplementary information, Fig. S8d (shNC group, 0 h). This error occurred because a repeated picture was used, which was due to our inadvertent preservation of representative picture of Supplementary information, Fig. S8i (vector group, 0 h). This experiment (wound healing assay) is a dynamic time-sequence observation process; we have meticulously reviewed the raw data and rectified the pictures by other sight field of the same experiment trial (0 h and 12 h as a set of data). This correction does not affect the quantification of the results or the conclusions of this study. The original Supplementary information, Fig. S8 has been corrected. We sincerely apologize for this oversight.

The original article has been corrected.

## Supplementary information

Supplementary Figure 8-Final updated

