# Supplementary material for: The rapid proximity labeling system PhastID identifies ATP6AP1 as an unconventional GEF for Rheb
Source: Cell Res. 2024 Mar 6;34(5):355–69. doi: 10.1038/s41422-024-00938-z (PMC11061317; doi:10.1038/s41422-024-00938-z)
Supplement: Supplementary file 1 — Supplementary information, Fig. S1 [file 41422_2024_938_MOESM1_ESM.pdf]

Supplementary information, Fig. S1

a

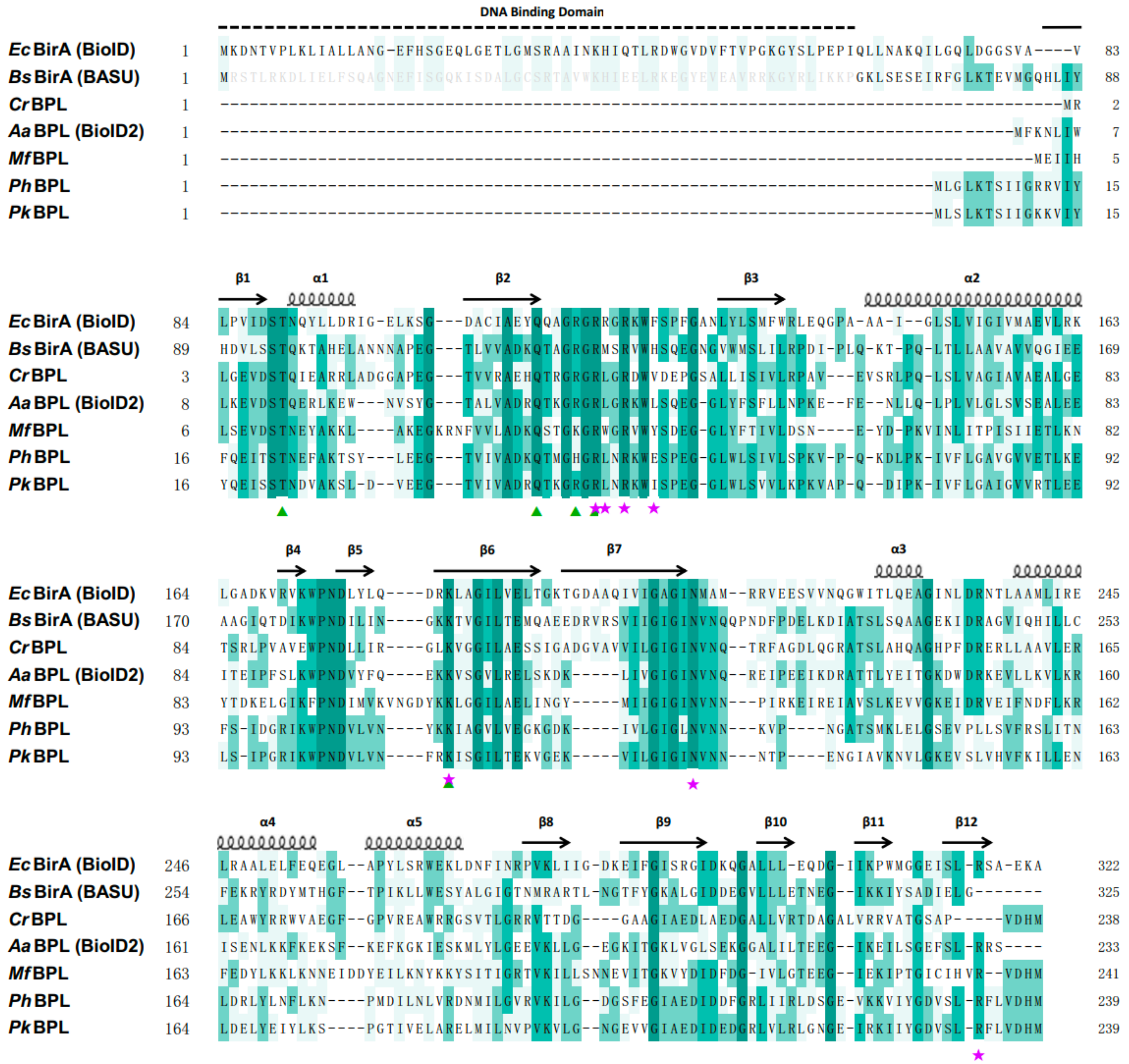

Supplementary information, Fig. S1

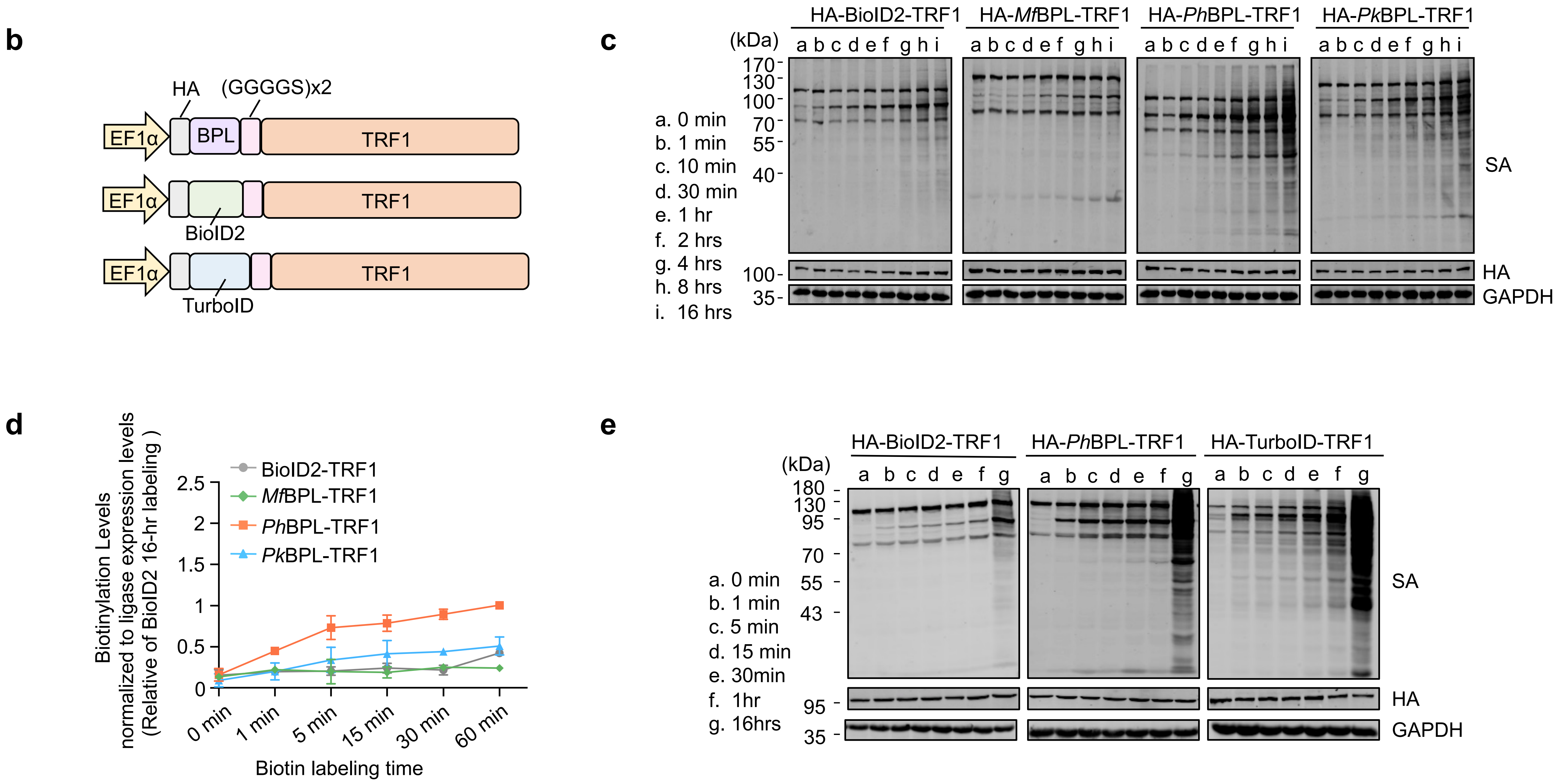

Supplementary information, Fig. S1

f

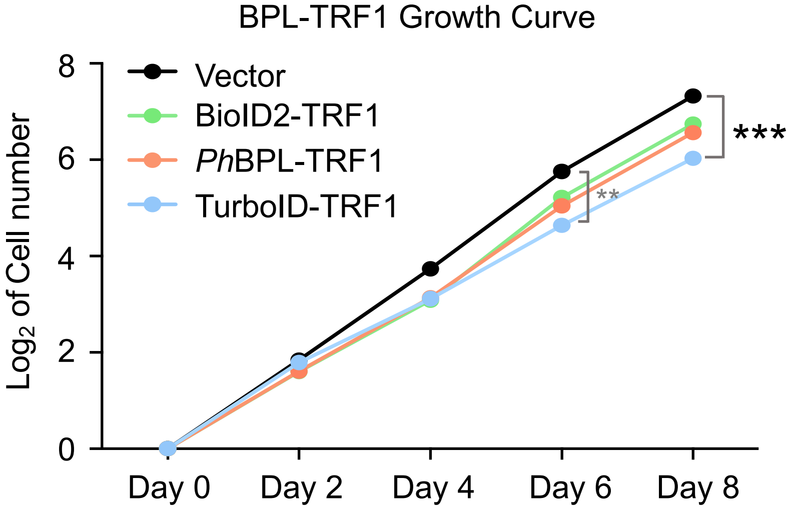

g

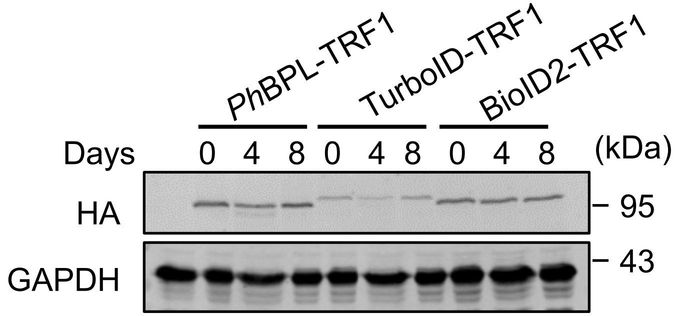

Supplementary information, Fig. S1. PhastID identifies TRF1-proximal proteomes.

a, Sequence alignment of the biotin ligases tested in this study compared with previously reported BPLs. Purple stars indicate combination with adenosine. Green triangles indicate combination with biotin. **b**, Diagram of the sequence elements in the vectors for BPL-TRF1 fusion protein expression. **c-d**, HeLa cells expressing the indicated HA-Flag-tagged TRF1-BPL fusion proteins were used for biotin labeling test and examined at various time points following biotin addition by western blotting using Streptavidin Protein DyLight™ 680 (SA), and anti-HA and GAPDH antibodies. The stable cell lines were cultured in dialyzed serum for 3 days, and then adding biotin (50 μM) labeling for indicated time (c). Total streptavidin signals from each lane were divided by the corresponding HA signals and then normalized to the 16-hour BioID2 sample as appropriate. Data from the first hour were plotted (d). **e**, HeLa cells expressing the indicated HA-Flag-tagged BPL-TRF1 fusion proteins were used for biotin labeling test and examined at various time points following biotin addition by western blotting. The stable cell lines were cultured in dialyzed serum for 3 days, and then adding biotin (50 μM) labeling for indicated time. Streptavidin Protein DyLight^{TM} 680, anti-HA and GAPDH antibodies were used for western blot. **f-g**, HeLa cells stably expressing vector alone or the indicated BPL-TRF1 fusion proteins were cultured in appropriate antibiotics for 7 days. Day 0 refers to the day that the stable cell line was established following antibiotic selection. Cells were maintained in complete media with biotin supplementation during these processes. Cell number (f) and protein expression (g) were determined at the indicated time points. Log2 transformed cell numbers were plotted using GraphPad Prism (n=3 biological repeats). Statistical significance was determined using the two-tailed Student's *t*-test, $^{**}p<0.01$, $^{***}p<0.001$.
