# Supplementary material for: The rapid proximity labeling system PhastID identifies ATP6AP1 as an unconventional GEF for Rheb
Source: Cell Res. 2024 Mar 6;34(5):355–69. doi: 10.1038/s41422-024-00938-z (PMC11061317; doi:10.1038/s41422-024-00938-z)
Supplement: Supplementary file 2 — Supplementary information, Fig. S2 [file 41422_2024_938_MOESM2_ESM.pdf]

Supplementary information, Fig. S2

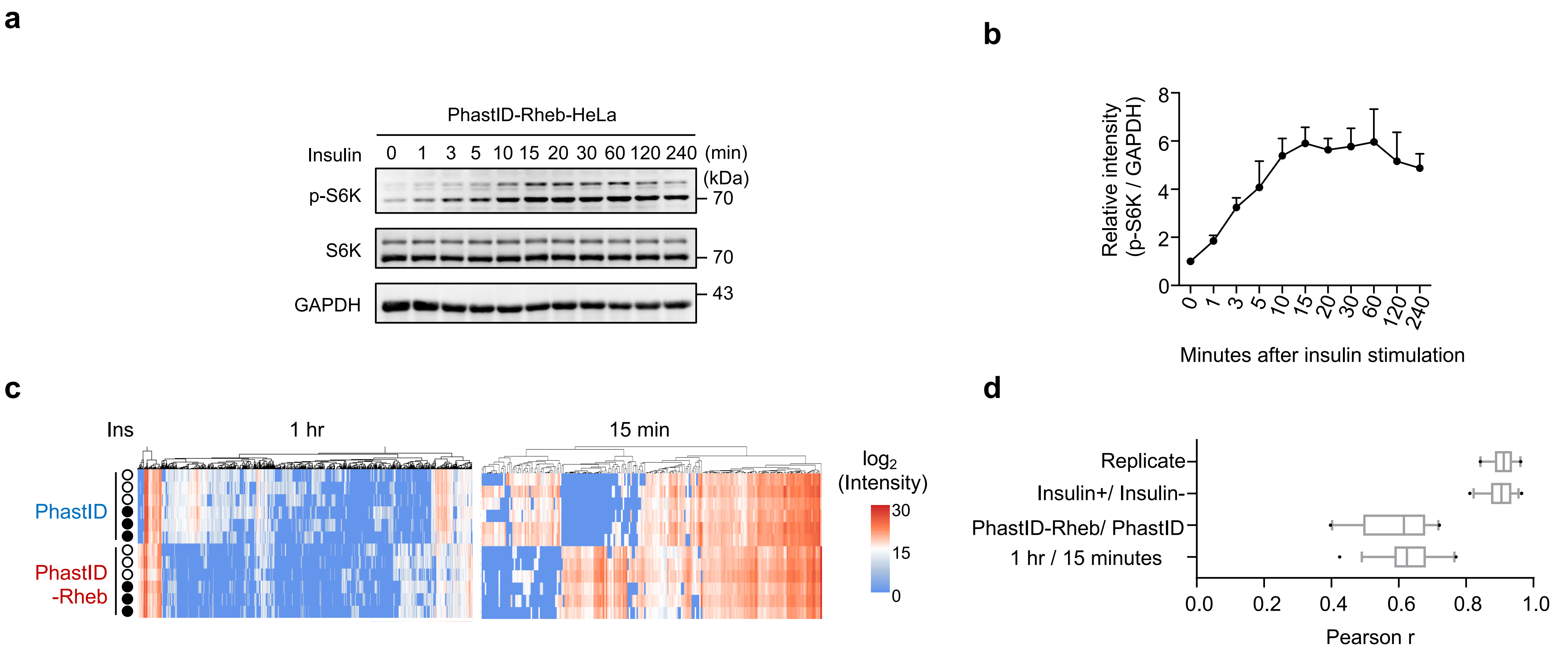

Supplementary information, Fig. S2

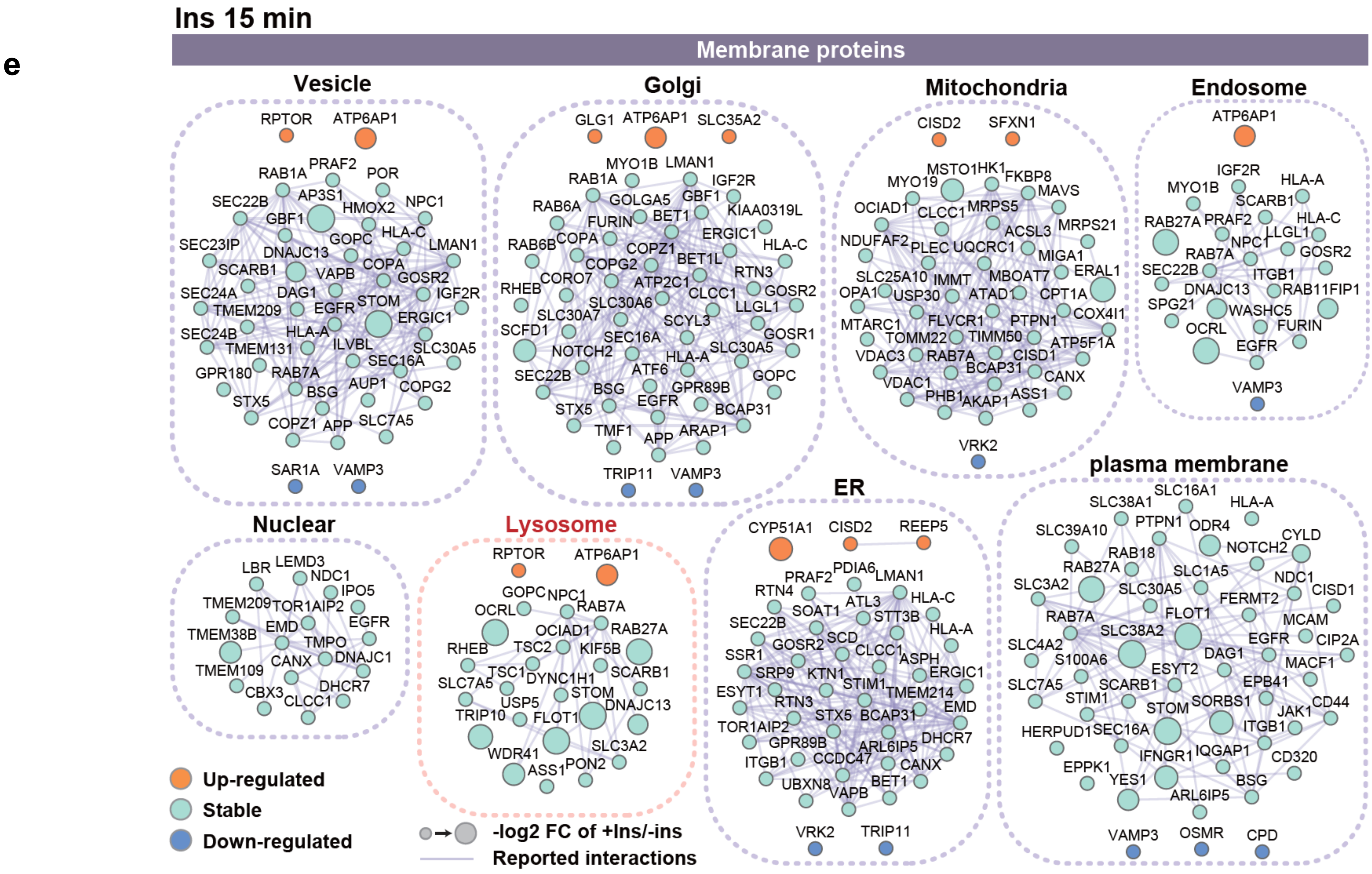

Supplementary information, Fig. S2

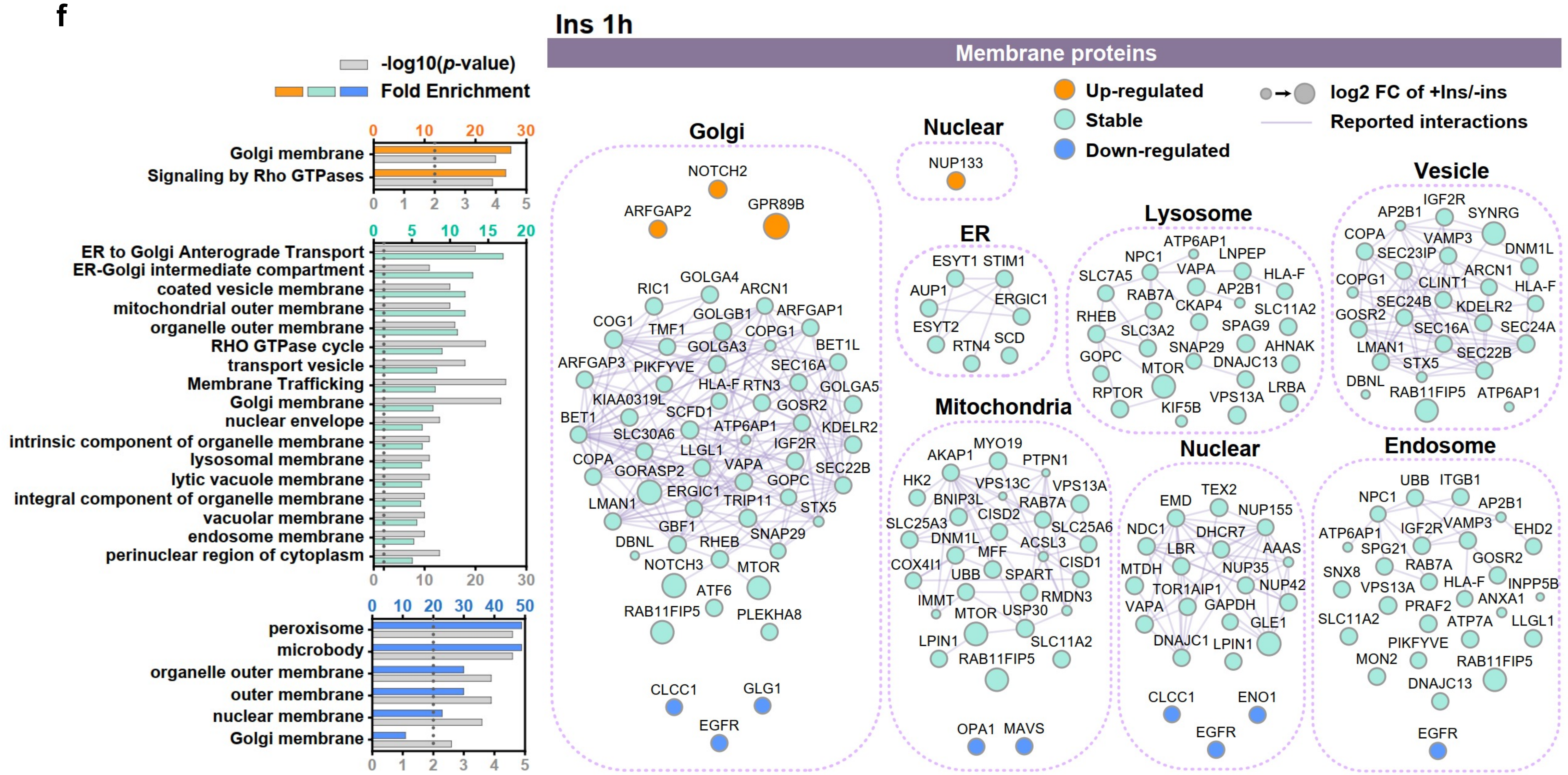

Supplementary information, Fig. S2. The proximal proteome of Rheb is revealed by PhastID.

a-b, HeLa cells stably expressing PhastID-Rheb were serum-deprived for 16 hours before the addition of insulin (0.9 µM). Cells collected at different time points were immunoblotted for the level of S6K phosphorylation at the Thr421/Ser424 site (p-S6K) (a). p-S6K signal intensities were normalized to total S6K and GAPDH signals and plotted in (b) as mean ± s.e.m (n=3 independent experiments). **c**, Heatmap of the PhastID-Rheb datasets compared to PhastID alone. Each group contained three independent repeats. **d**, Pearson correlation coefficient of PhastID-Rheb (- /+ insulin) and PhastID (- /+ insulin) results. **e**, Rheb-interacting proteins from the 15-minute treatment group were divided based on their subcellular localization. Circle size reflects the $-\log_2$(Fold change) of FOT changes after insulin treatment. Candidates are color coded to denote interactions that were unaffected (stable) or affected (up- or down-regulated) by insulin treatment. More than 1.5-fold change was defined as up-regulated group. Fold change less than 0.67-fold was defined as down-regulated group. Fold change between 0.67- and 1.5-fold was defined as stable group. **f**, Enriched candidates from the 1-hour insulin stimulation datasets were grouped into categories of up-regulated (insulin treated group divided by no insulin-treated group, fold change more than 1.5-fold), down-regulated (insulin treated group divided by no insulin-treated group fold change less than 0.67-fold), or stable interactions following insulin stimulation (insulin treated group divided by no insulin-treated group, fold change between 0.67- and 1.5-fold), and plotted based on cellular localization and relative abundance analysis (right) or GO analysis (left).
