# Supplementary material for: The rapid proximity labeling system PhastID identifies ATP6AP1 as an unconventional GEF for Rheb
Source: Cell Res. 2024 Mar 6;34(5):355–69. doi: 10.1038/s41422-024-00938-z (PMC11061317; doi:10.1038/s41422-024-00938-z)
Supplement: Supplementary file 3 — Supplementary information, Fig. S3 [file 41422_2024_938_MOESM3_ESM.pdf]

Supplementary information, Fig. S3

a

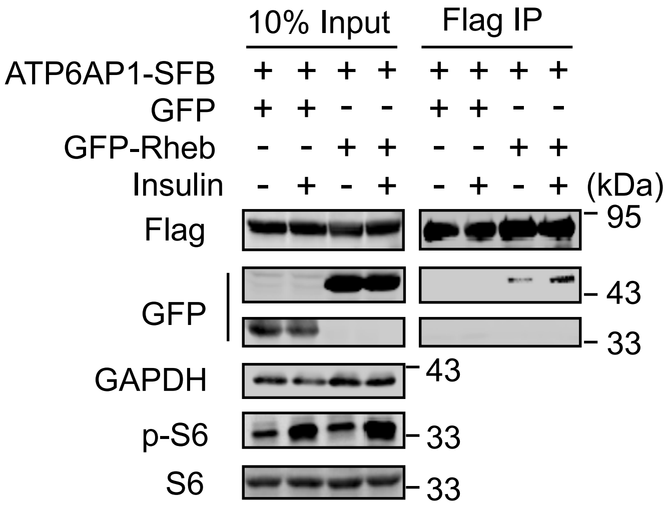

b

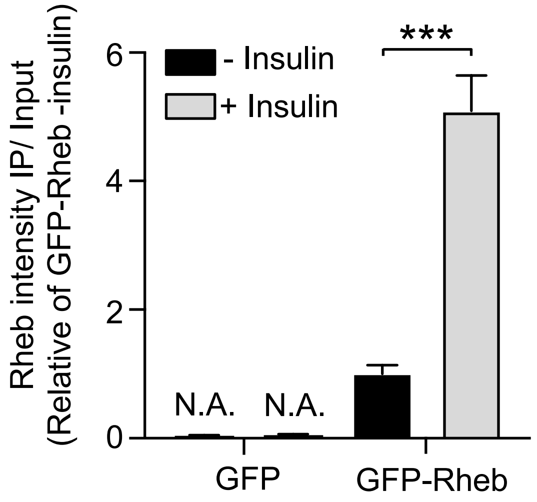

c

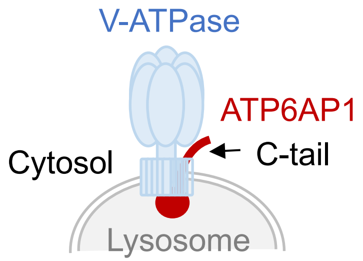

Supplementary information, Fig. S3

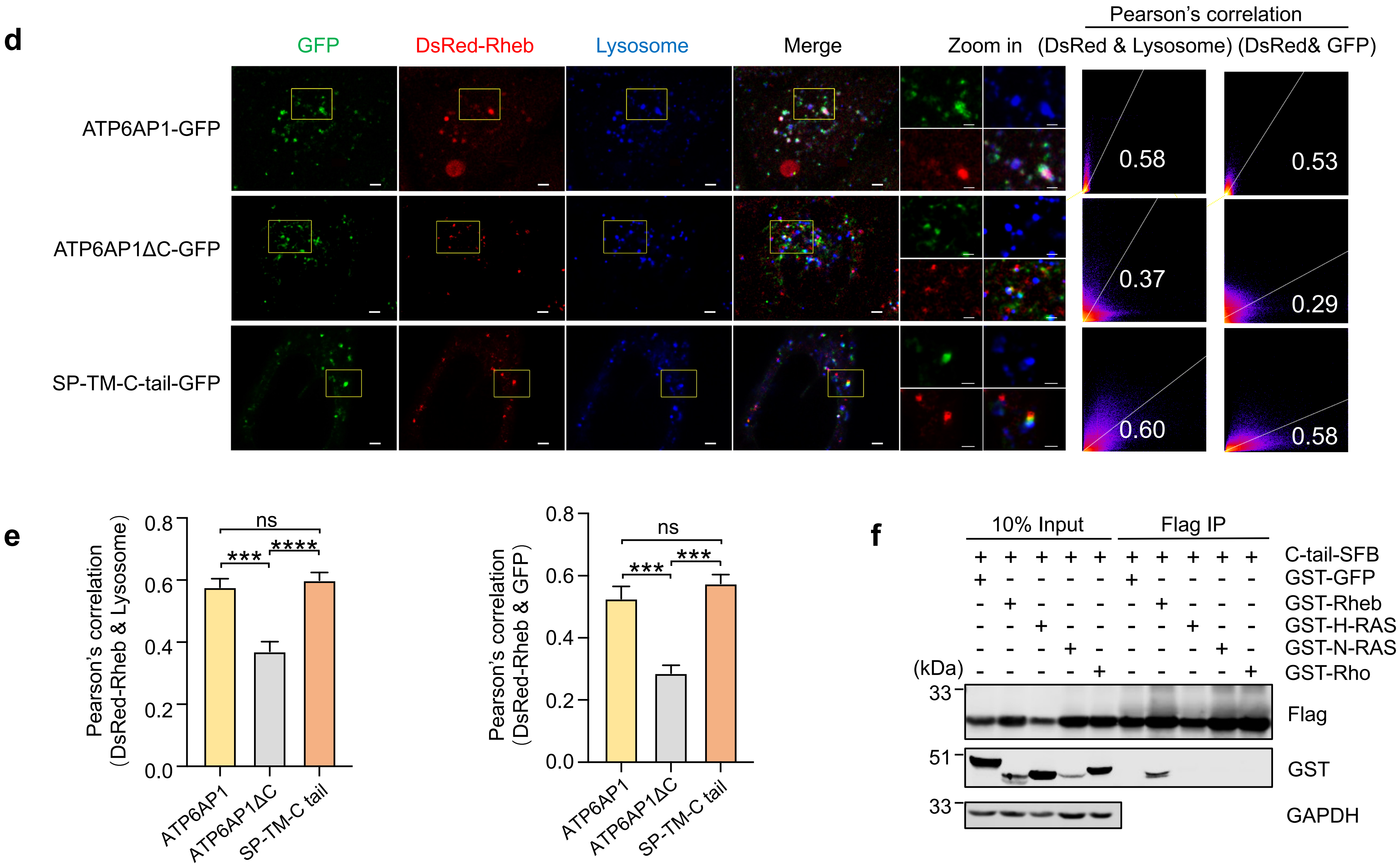

Supplementary information, Fig. S3. ATP6AP1 interacts with Rheb through C-tail.

a-b, HEK293 cells stably co-expressing ATP6AP1-SFB and GFP-Rheb were serum starved and then treated with insulin for 15 minutes. The cells were then collected for anti-Flag IP and immunoblotting as indicated. Compared with GFP-Rheb without insulin treatment group. Statistical significance was determined using the two-way ANOVA followed by Dunnett's multiple comparisons test, $***p<0.001$. N.A., not available. **c**, ATP6AP1 sits at the bottom of lysosomal V-ATPase, with its C tail extending outside of the lysosome and into the cytosol. **d-e**, HeLa cells stably co-expressing DsRed-Rheb with GFP-tagged full-length ATP6AP1 or its deletion mutants (SP-TM-C-tail or ATP6AP1-ΔC) were immunostained with antibodies against the lysosomal marker LAMP1 (blue). Scale bar: 5 µm (d). Boxed regions are enlarged in zoom panels. Pearson's correlation coefficient of co-localization of DsRed-Rheb with Lysosome (left) or DsRed-Rheb with full-length ATP6AP1 or its deletion mutants (right) using 2D intensity histogram output of coloc2 analysis performed with Fiji/ImageJ. Statistical significance was determined using the two-way ANOVA followed by Dunnett's multiple comparisons test, $***p<0.001$, $****p< 0.0001$. ns, not significant. **f**, HEK293T cells were transiently transfected with SFB-tagged C-tail(30) of ATP6AP1 and GST-tagged GFP, Rheb, H-RAS, N-RAS or Rho, respectively. The cells were then collected for immunoprecipitation (IP) with anti-Flag antibody coupled agarose beads and immunoblotting with the GST antibodies, GAPDH was served as a loading control.
