# Supplementary material for: The rapid proximity labeling system PhastID identifies ATP6AP1 as an unconventional GEF for Rheb
Source: Cell Res. 2024 Mar 6;34(5):355–69. doi: 10.1038/s41422-024-00938-z (PMC11061317; doi:10.1038/s41422-024-00938-z)
Supplement: Supplementary file 4 — Supplementary information, Fig. S4 [file 41422_2024_938_MOESM4_ESM.pdf]

Supplementary information, Fig. S4

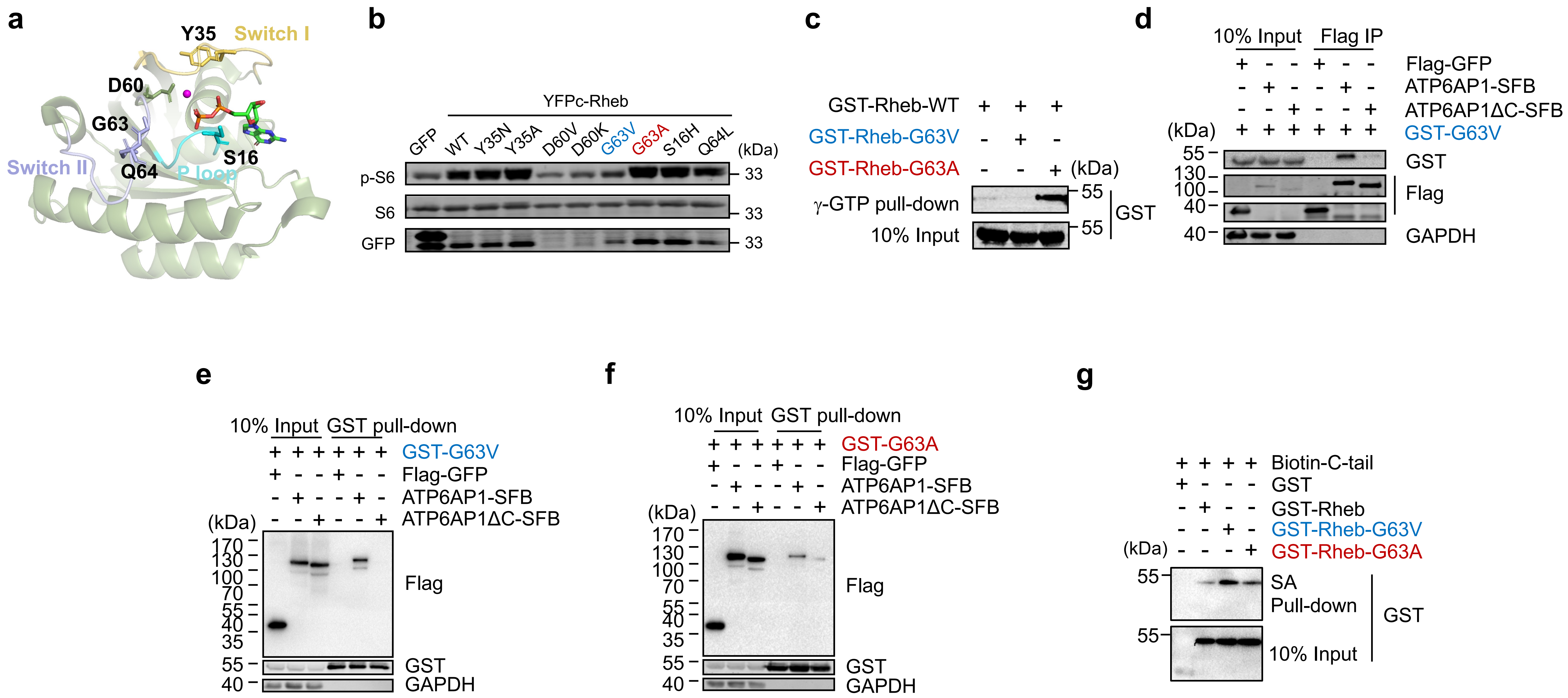

Supplementary information, Fig. S4

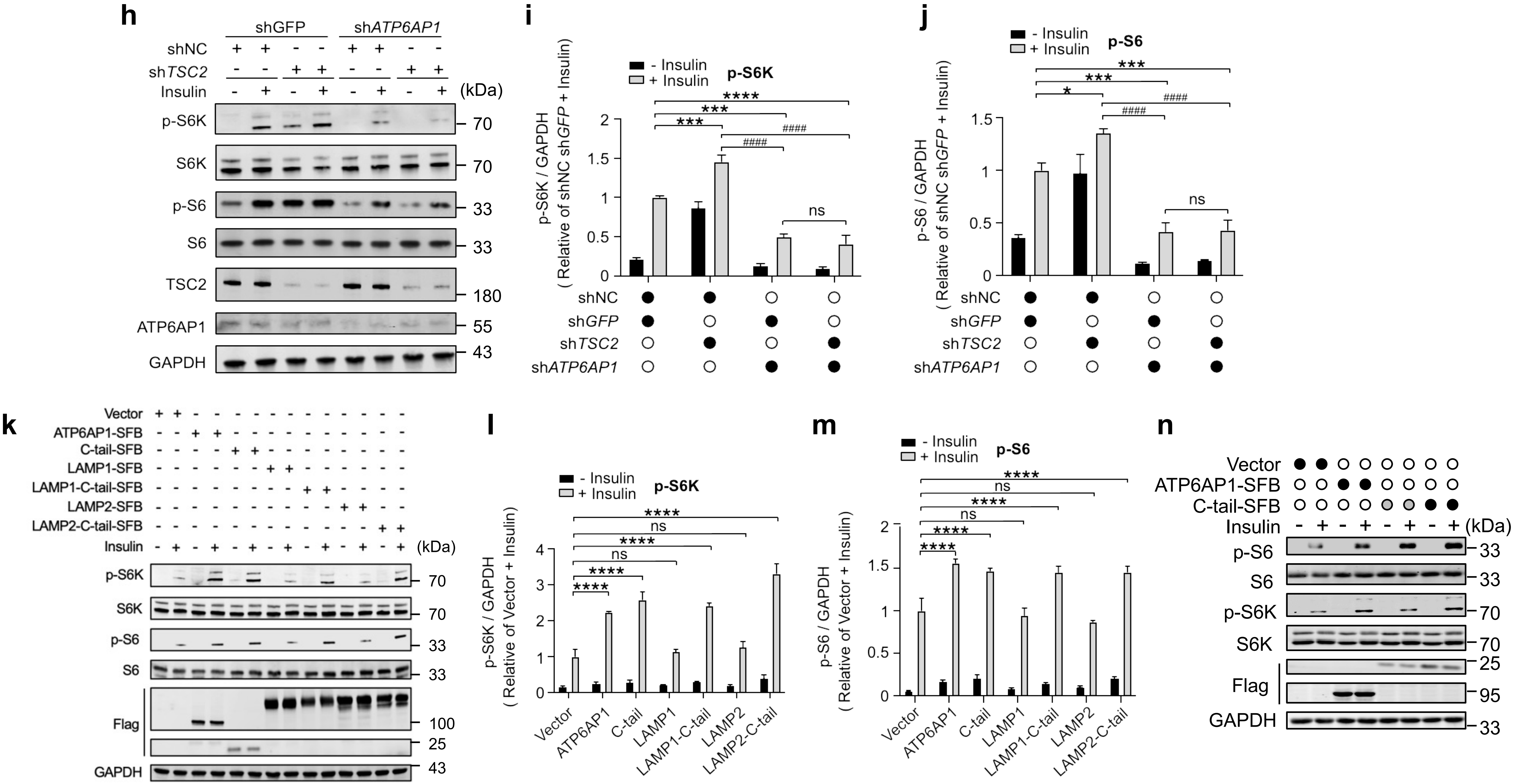

Supplementary information, Fig. S4. ATP6AP1 preferentially binds with inactive form of Rheb.

a, The overall structure of GDP-bound WT Rheb (PDB code 1XTQ). The switch I region is in yellow, the switch II region in lavender, and the P loop region in cyan. Mg^{2+} is shown as a magenta-colored sphere, and GDP as a ball-and-stick model in green. Residues reported to influence nucleotide preference are highlighted. **b**, HeLa cells stably expressing YFPc-tagged wildtype or mutant Rheb were analyzed by immunoblotting. An anti-GFP antibody that recognizes the YFPc fragment was used to detect exogenously expressed Rheb. **c**, Recombinant GST-tagged wildtype and mutant Rheb proteins were incubated with γ-GTP beads for *in vitro* pulldown followed by western blotting. **d**, HEK293T cells transiently co-expressing GST-tagged Rheb-G63V and SFB-tagged full-length or C-tail deletion mutant of ATP6AP1 were harvested for anti-Flag IP and immunoblotting. **e-f**, HEK293T cells transiently co-expressing SFB-tagged full-length or C-tail deletion mutant of ATP6AP1 together with GST-tagged Rheb-G63V (e) or Rheb-G63A (f) were harvested for GST pulldown and immunoblotting. **g**, A synthetic biotinylated peptide containing the last 30 residues of ATP6AP1 (C-tail 30) was incubated with bacterially purified recombinant GST-tagged Rheb, Rheb-G63V, or Rheb-G63A. The bound proteins were brought down using streptavidin (SA) beads, resolved by SDS-PAGE, and blotted as indicated. **h-j**, Ctrl or *TSC2* double-knockdown HeLa cells expressing Ctrl or *ATP6AP1* shRNAs were starved of serum for 16 hours and re-stimulated with 0.9 μM insulin for 15 minutes before immunoblotting. Intensity values for p-S6K/S6 were similarly processed as above and graphed as mean ± s.e.m. (n=3). Statistical significance was determined using the two-way ANOVA followed by Tukey's multiple comparisons test, *$p<0.05$, ***$p<0.001$, ****$p<0.0001$, ####$p<0.0001$. ns, not significant. shNC plus sh*GFP* with insulin treatment / shNC plus sh*TSC2* with insulin treatment / sh*GFP* plus sh*ATP6AP1* with insulin treatment served as a negative control.

Supplementary information, Fig. S4. ATP6AP1 preferentially binds with inactive form of Rheb.

k-m, HeLa cells transiently expressing SFB-tagged ATP6AP1, C-tail, LAMP1, LAMP1-C-tail, LAMP2, LAMP2-C-tail (LAMP1-C-tail and LAMP2-C-tail refers to the hybrid proteins that the C-terminal tail of LAMP1 and LAMP2 were replaced with ATP6AP1 C-tail (30), respectively) were serum-starved followed by 15-minute treatment with 0.9 μM insulin. The cells were then collected for western analysis using the indicated antibodies. Vector with insulin treatment served as a negative control. GAPDH was used as a loading control (k). Intensity values were obtained using ImageJ. p-S6K (l) and p-S6 (m) signals were normalized to total S6K/S6 as well as GAPDH and graphed as mean ± s.e.m. (n=3). Statistical significance was determined using the two-way ANOVA followed by Sidak's multiple comparisons test, ****p<0.0001, ns, not significant. **n**, HeLa cells were transfected with different amounts of constructs encoding SFB-tagged C-tail of ATP6AP1. Vector alone and SFB-tagged full-length ATP6AP1 served as negative and positive controls respectively. The cells were serum starved, treated with insulin (0.9 μM) for 15 minutes, and then collected for western blotting. The depth of the circle color represents different doses. Dark circles represent high doses of 4 μg of indicated plasmids per well for transfection, while gray circles denote 2 μg of plasmids. The hollow white circles indicate no addition of relative plasmid.
