# Supplementary material for: The rapid proximity labeling system PhastID identifies ATP6AP1 as an unconventional GEF for Rheb
Source: Cell Res. 2024 Mar 6;34(5):355–69. doi: 10.1038/s41422-024-00938-z (PMC11061317; doi:10.1038/s41422-024-00938-z)
Supplement: Supplementary file 5 — Supplementary information, Fig. S5 [file 41422_2024_938_MOESM5_ESM.pdf]

Supplementary information, Fig. S5

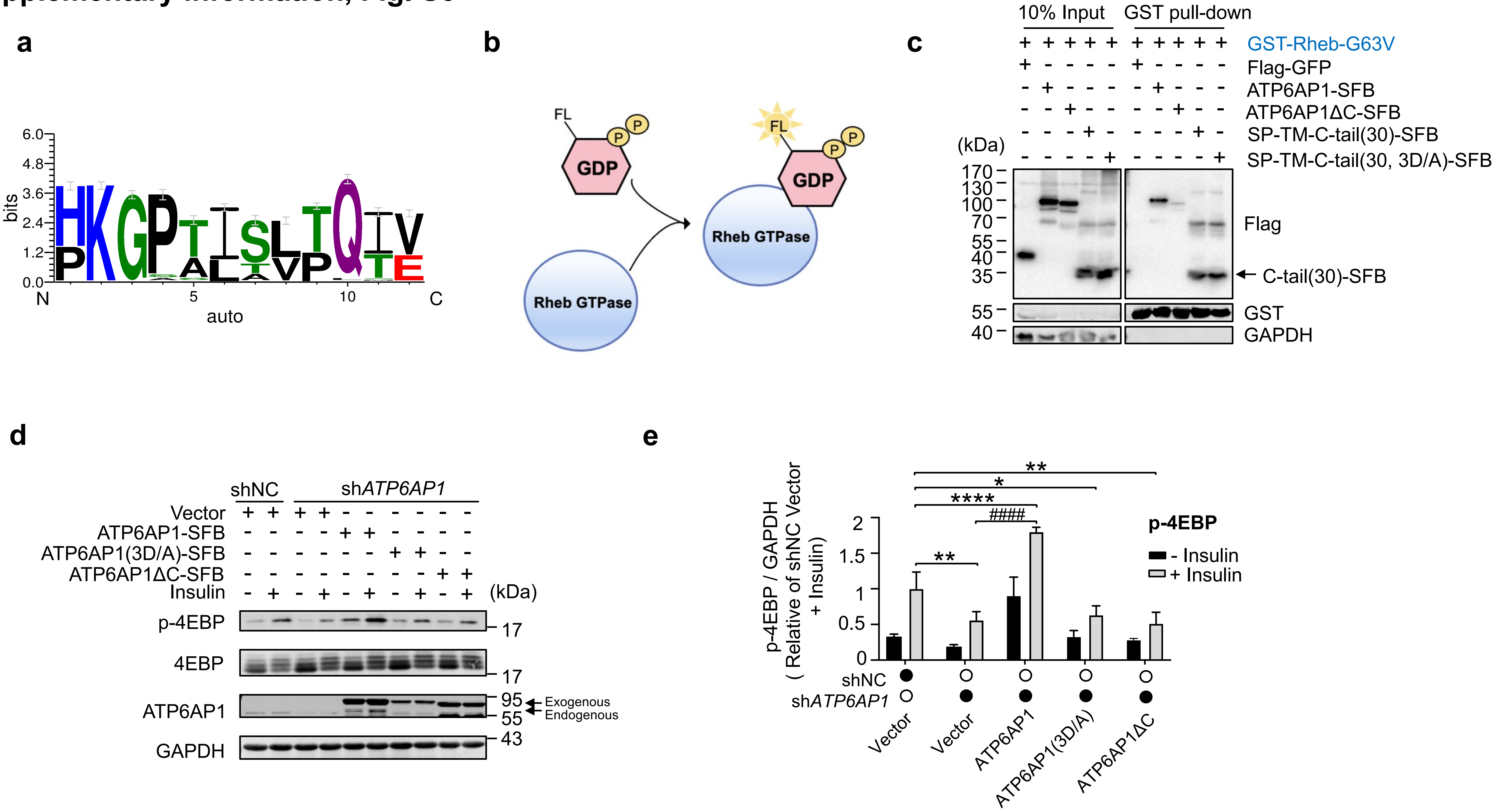

Supplementary information, Fig. S5

f

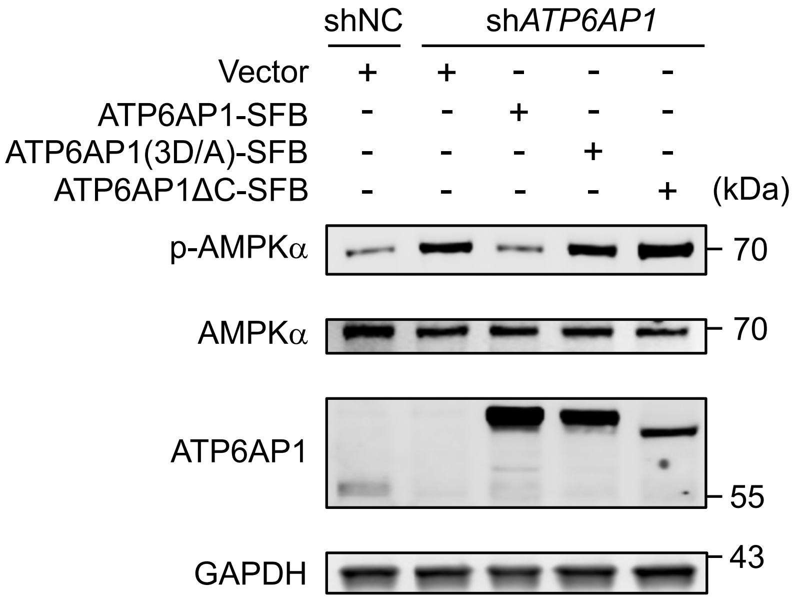

g

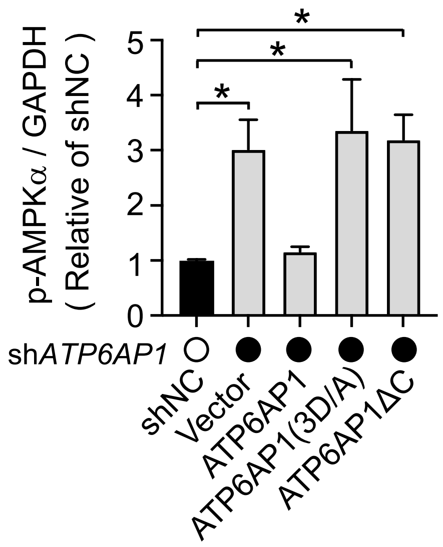

Supplementary information, Fig. S5

h

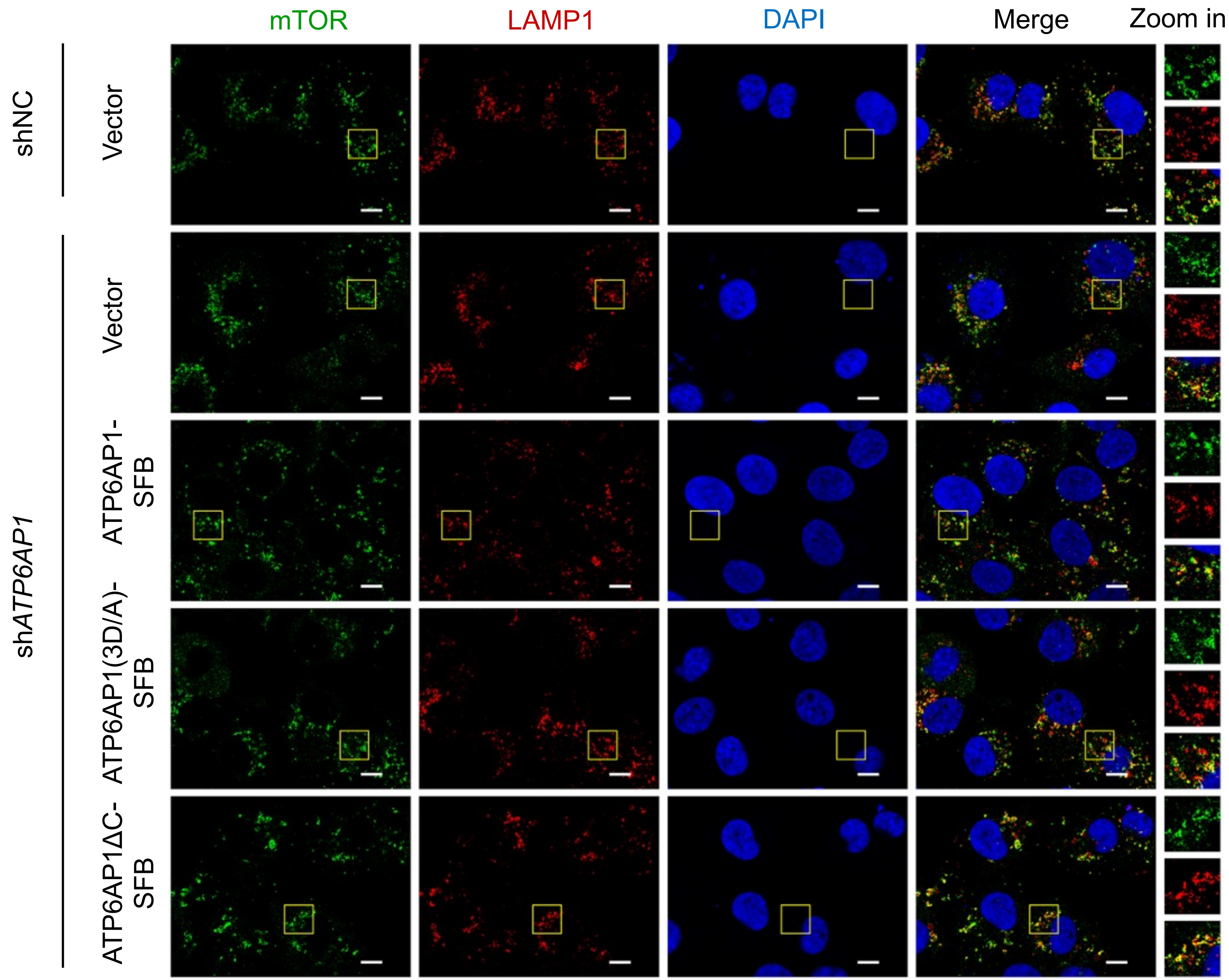

i

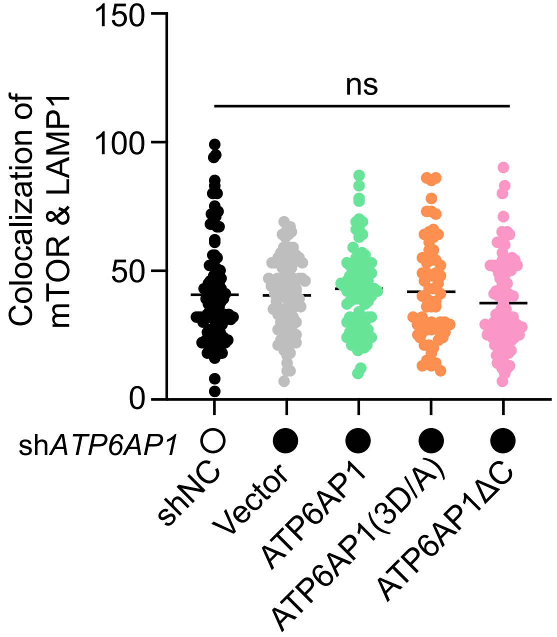

Supplementary information, Fig. S5

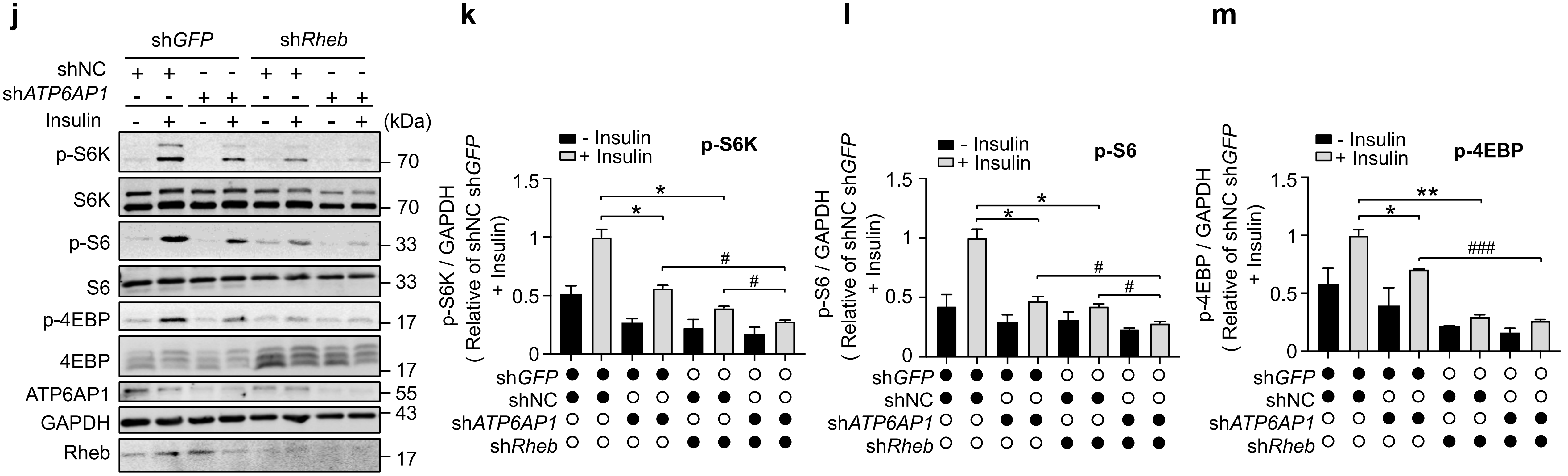

Supplementary information, Fig. S5

n

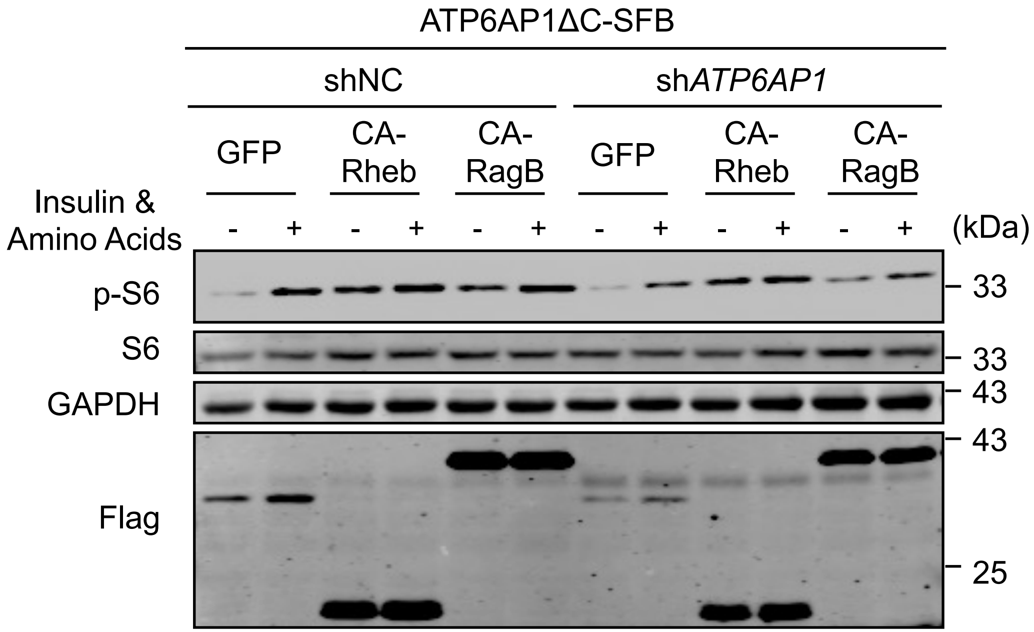

o

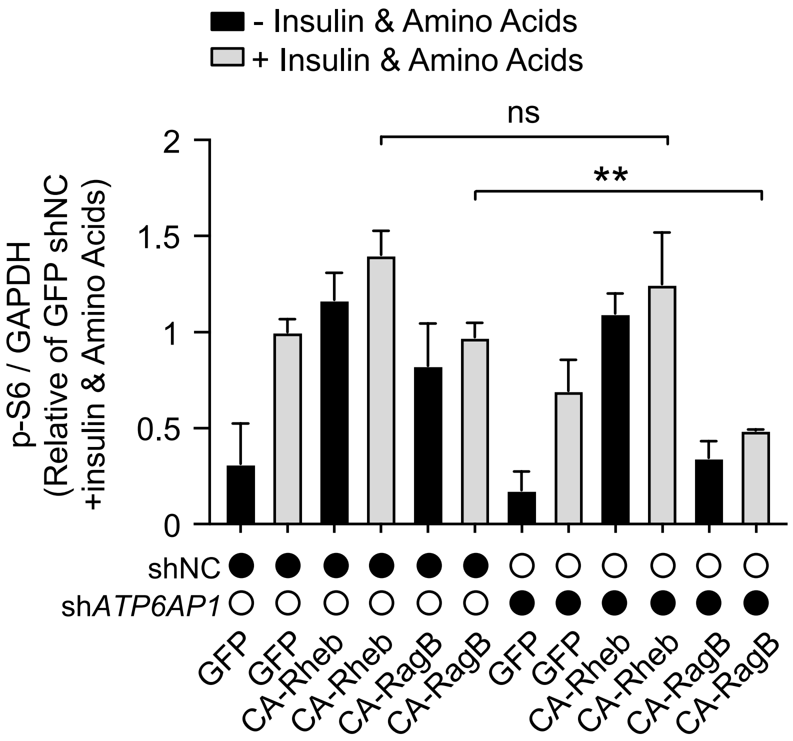

p

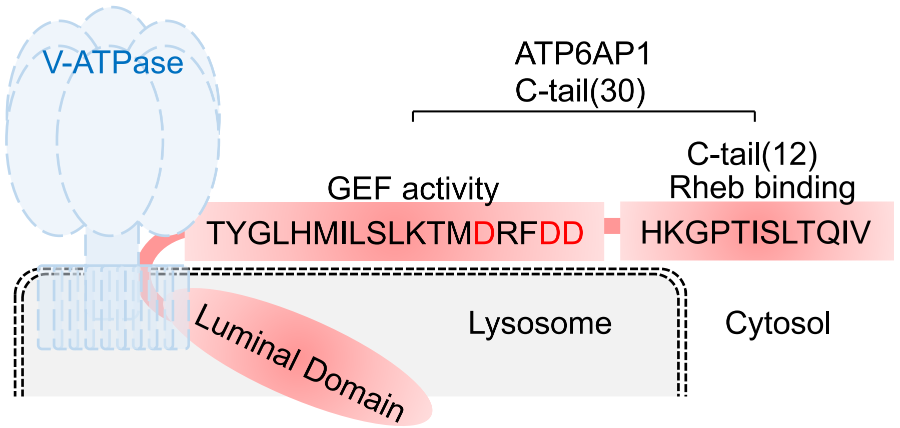

Supplementary information, Fig. S5. The GEF domain and Rheb-binding domain of ATP6AP1 may be separated.

a, The alignment of conserved sequence of C-tail (12). **b**, In this binding assay using the green fluorescent BIODIPY FL dye, fluorescence signal recovery upon binding of the quenched fluorophore-conjugated GTP/GDP analogs to G proteins enables real-time monitoring of G protein binding to GTP/GDP. **c**, HEK293T cells transiently co-expressing GST-G63V and the indicated SFB-tagged full-length or mutant ATP6AP1 were used for GST pulldown and western blotting. Flag-tagged GFP served as a negative control. **d-e**, HeLa cells transiently expressing the indicated SFB-tagged sequences were serum starved and then treated with insulin (0.9 µM) for 15 minutes before western blot analysis. Stable *ATP6AP1*-knockdown HeLa cells that also expressed RNAi-resistant wildtype or mutant ATP6AP1 (d). Intensity values for p-4EBP (e) were similarly processed as above and graphed as mean ± s.e.m (n=3). Statistical significance was determined using the two-way ANOVA followed by Dunnett's multiple comparisons test, *p<0.05, **p<0.01, ****p<0.0001, ####p<0.001. shNC with insulin treatment and sh*ATP6AP1* with insulin treatment separated served as a negative control. **f-g**, Stable *ATP6AP1*-knockdown HEK293T cells cells transiently expressing with indicated SFB-tagged full-length or mutant ATP6AP1 were immunoblotted to detect the indicated proteins and phospho-proteins (f). Intensity values for p-AMPKα (g) were similarly processed as above and graphed as mean ± s.e.m. (n=3). Statistical significance was determined using the two-way ANOVA followed by Tukey's multiple comparisons test, *p<0.05. shNC served as a control. **h-i**, MDA231 cells stably expressing with indicated SFB-tagged full-length or mutant ATP6AP1 were immunostained with antibodies against the lysosomal marker LAMP1 (red) and mTOR (green), co-stained with DAPI (blue) for DNA content. Scale bar: 5 µm (h). Boxed regions are enlarged in zoom panels. Quantification of the co-localization between mTOR and LAMP1 was shown in i. Statistical significance was determined using the two-way ANOVA followed by Tukey's multiple comparisons test. ns, not significant.

Supplementary information, Fig. S5. The GEF domain and Rheb-binding domain of ATP6AP1 may be separated.

j-m, Ctrl or *Rheb* double-knockdown HeLa cells expressing Ctrl or *ATP6AP1* shRNAs were starved of serum for 16 hours and re-stimulated with 0.9 μM insulin for 15 minutes before immunoblotting (j). Intensity values for p-S6K (k) /S6 (l) /4EBP (m) were similarly processed as above and graphed as mean ± s.e.m. (n=3). Statistical significance was determined using the two-way ANOVA followed by Dunnett's multiple comparisons test, *$p<0.05$, **$p<0.01$, #$p<0.05$, ###$p<0.001$. shNC plus sh*GFP* with insulin treatment / sh*ATP6AP1* plus sh*Rheb* with insulin treatment served as a control. **n-o**, ATP6AP1-ΔC cells expressing Ctrl or *ATP6AP1* shRNAs were transfected with constitutively active forms of Rheb (CA-Rheb) or RagB (CA-RagB) and immunoblotted to detect the indicated phospho-proteins (n). Intensity values for p-S6 (o) were similarly processed as above and graphed as mean ± s.e.m. (n=3). Statistical significance was determined using the two-way ANOVA followed by Dunnett's multiple comparisons test, **$p<0.01$. ns, not significant. shNC expressed with CA-RagB / CA-Rheb with insulin treatment served as controls. **p**, Distinct regions within the ATP6AP1 C-tail mediate GEF and Rheb-binding activities.
