# Supplementary material for: The rapid proximity labeling system PhastID identifies ATP6AP1 as an unconventional GEF for Rheb
Source: Cell Res. 2024 Mar 6;34(5):355–69. doi: 10.1038/s41422-024-00938-z (PMC11061317; doi:10.1038/s41422-024-00938-z)
Supplement: Supplementary file 6 — Supplementary information, Fig. S6 [file 41422_2024_938_MOESM6_ESM.pdf]

Supplementary information, Fig. S6

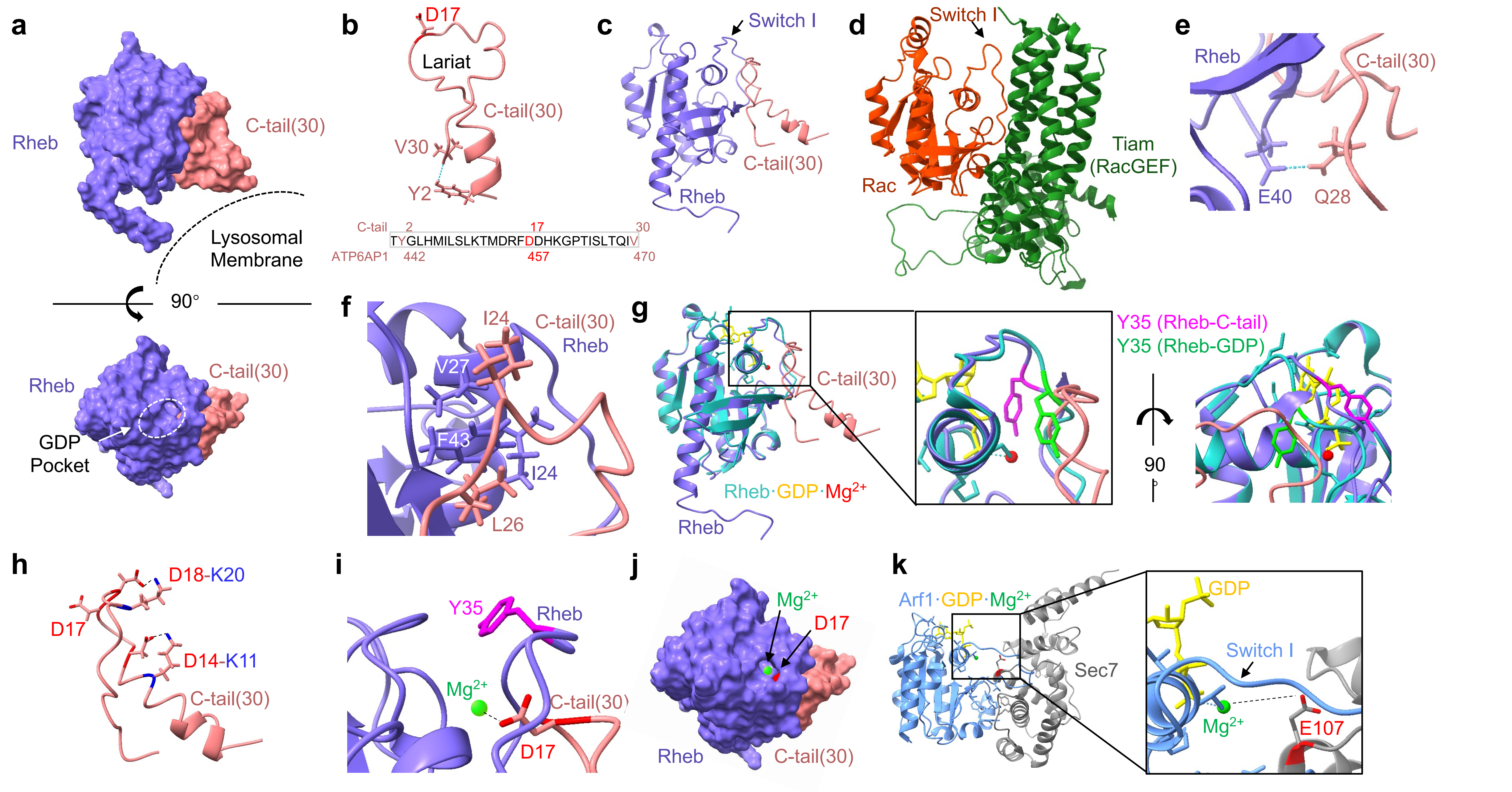

Supplementary information, Fig. S6. Predicted structure of Rheb and ATP6AP1 C-tail complex by AlphaFold-Multimer.

a, Structure of Rheb and C-tail(30) as modelled by AlphaFold-Multimer. The lipid-linked C-terminal of Rheb and the N-terminal of ATP6AP1 C-tail have a consistent orientation towards the lysosomal membrane. The complex interaction interface is located on the side of the GDP sitting pocket. **b**, The structural prediction shows that C-tail forms a lariat, and the C-terminal carboxyl group of V30 forms a hydrogen bond with the Y2 side chain. The amino acid sequence number of C-tail corresponds to its sequence number in full-length ATP6AP1. The numbers shown in this figure are the sequence numbers in C-tail. **c**, C-tail interacts with switch I region of Rheb. **d**, The structure of small G protein Rac and its GEF Tiam (PDB: 1FOE). Tiam also interacts with switch I region of Rab. **e**, Hydrogen bond tween Rheb E40 and C-tail Q28. **f**, C-tail TISLT motif binds with Rheb via hydrophobic interaction. **g**, Superposition of Rheb•GDP structure (PDB: 1XTQ, light blue) with predicted Rheb•C-tail complex (purple and pink). After binding to C-tail, there was a significant change in the position of Rheb Y35 residue. **h**, Intramolecular charge interactions may participate in stabilizing the C-tail structure and expose D17 on the other side. **i**, D17 residue, as an acidic finger, comes into contact with magnesium ion from within the switch I loop. **j**, D17 contacts with magnesium ion from the bottom of GDP pocket. **k**, The structure of small G protein Arf1 with its GEF Sec7 domain of ARNO (PDB: 1R8Q). Sec7 binds with Arf1 switch I region and uses its E107 as acidic finger to touch magnesium ion.
