# Supplementary material for: The rapid proximity labeling system PhastID identifies ATP6AP1 as an unconventional GEF for Rheb
Source: Cell Res. 2024 Mar 6;34(5):355–69. doi: 10.1038/s41422-024-00938-z (PMC11061317; doi:10.1038/s41422-024-00938-z)
Supplement: Supplementary file 7 — Supplementary information, Fig. S7 [file 41422_2024_938_MOESM7_ESM.pdf]

Supplementary information, Fig. S7

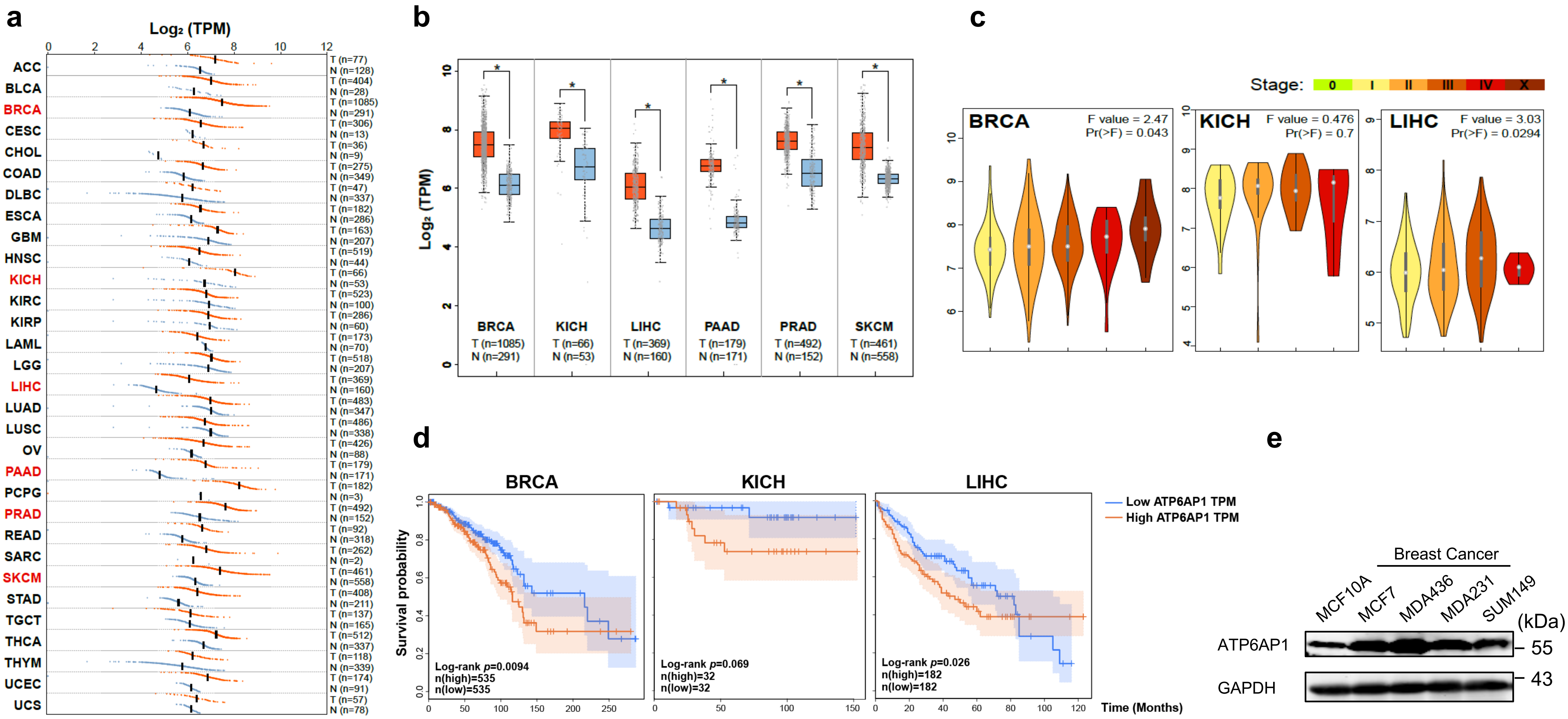

Supplementary information, Fig. S7

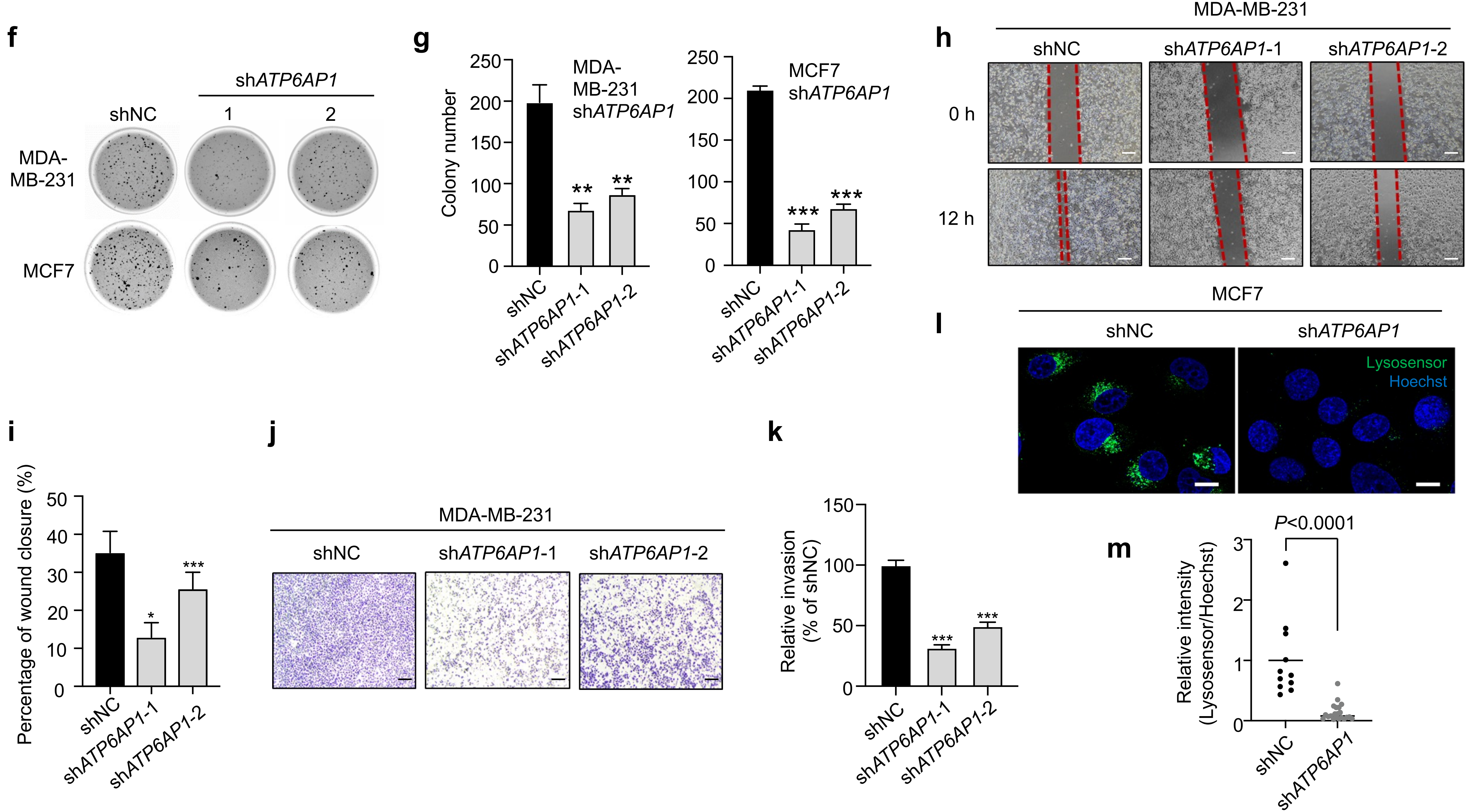

Supplementary information, Fig. S7. Targeting ATP6AP1 inhibits cancer cell growth and migration.

a-c, Comparison of ATP6AP1 mRNA levels in various cancer types vs. their normal tissue counterparts by the GEPIA online tool using TCGA and GTEx RNA-seq datasets (a). ATP6AP1 transcripts per million (TPM) was plotted in red for tumors (T) and blue for normal tissues (N). Cancers that show significantly different expression are marked in red and further graphed in box (b) and stage (c) plots. **d**, Survival probability of patients with BRCA, KICH, and LIHC was plotted based on ATP6AP1 expression. **e**, ATP6AP1 protein levels in several breast cancer cell lines and the non-tumorigenic breast epithelial cell line MCF10A were determined by western blotting. GAPDH was used as a loading control. **f-g**, MDA-MB-231 cells stably expressing different shRNAs targeting *ATP6AP1* were seeded in soft agar (top 0.35%, bottom 0.6%) and cultured for 14 days. Colonies were stained with MTT for one hour and visualized under microscopy (f). The data were quantified and presented (g) as mean ± s.e.m (n=3, $^{**}p<0.01$, $^{***}p<0.001$). shNC, non-targeting shRNA. **h-k**, MDA-MB-231 cells stably expressing different shRNAs targeting *ATP6AP1* were examined in wound healing (h-i) and transwell (j-k) assays. Scale bar: 100 μm (h, j). The data were quantified and presented (i, k) as mean ± s.e.m (n=3, $^{*}p<0.05$, $^{***}p<0.001$). shNC, non-targeting shRNA. **l-m**, MCF7 cells stably expressing shRNAs targeting *ATP6AP1* were labeled with the Lysosensor dye to assess lysosomal pH (l). Hoechst 33342 (C1022, Beyotime) was used to stain the nuclei. Scale bar: 20 μm. Relative Lysosensor intensity was graphed (m) as mean ± s.e.m (n=3 biological repeats). Statistical significance was determined using the two-tailed Student's t-test, shNC, non-targeting shRNA.
