# Supplementary material for: The rapid proximity labeling system PhastID identifies ATP6AP1 as an unconventional GEF for Rheb
Source: Cell Res. 2024 Mar 6;34(5):355–69. doi: 10.1038/s41422-024-00938-z (PMC11061317; doi:10.1038/s41422-024-00938-z)
Supplement: Supplementary file 8 — Supplementary information, Fig. S8 [file 41422_2024_938_MOESM8_ESM.pdf]

Supplementary information, Fig. S8

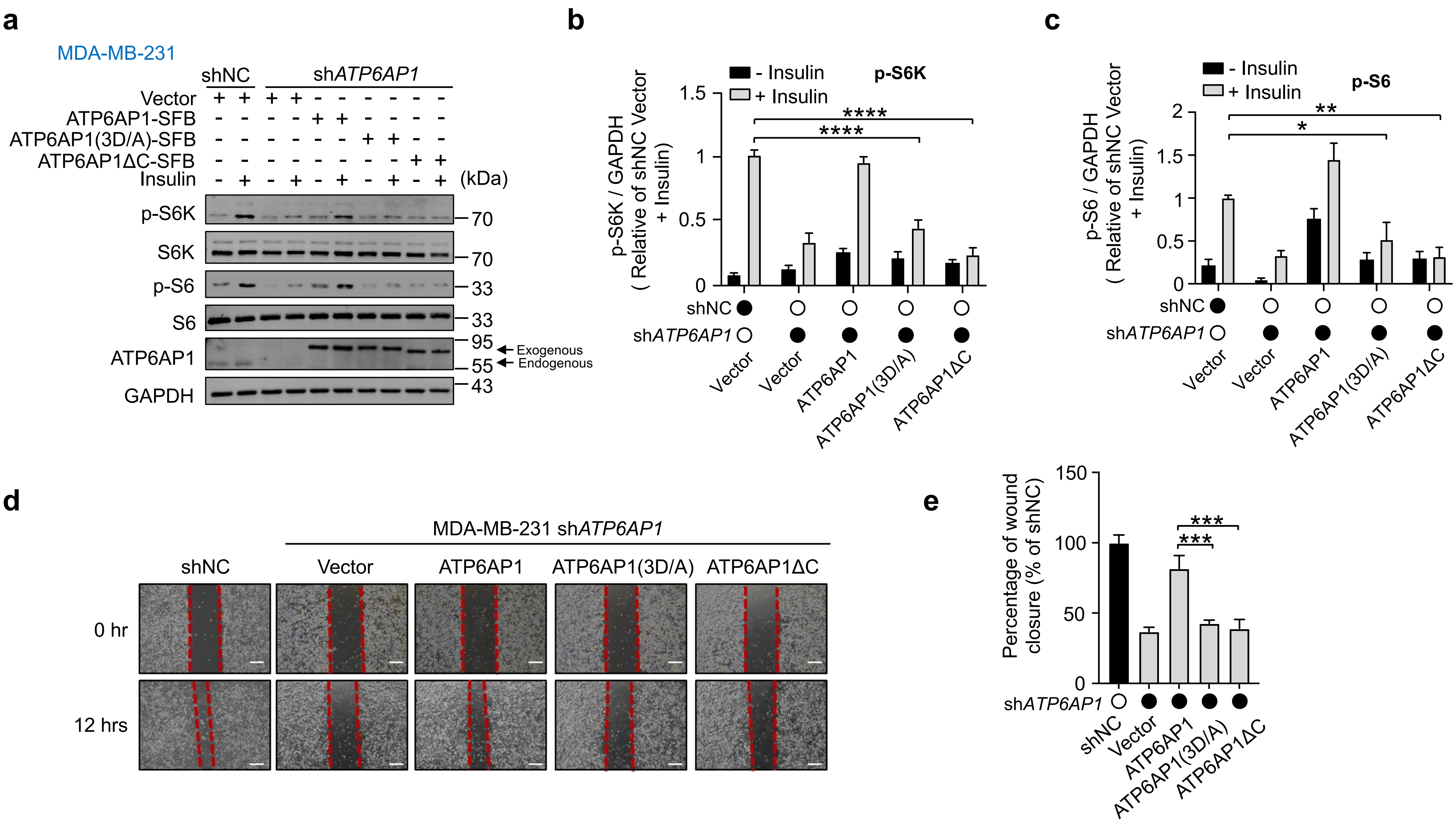

Supplementary information, Fig. S8

f

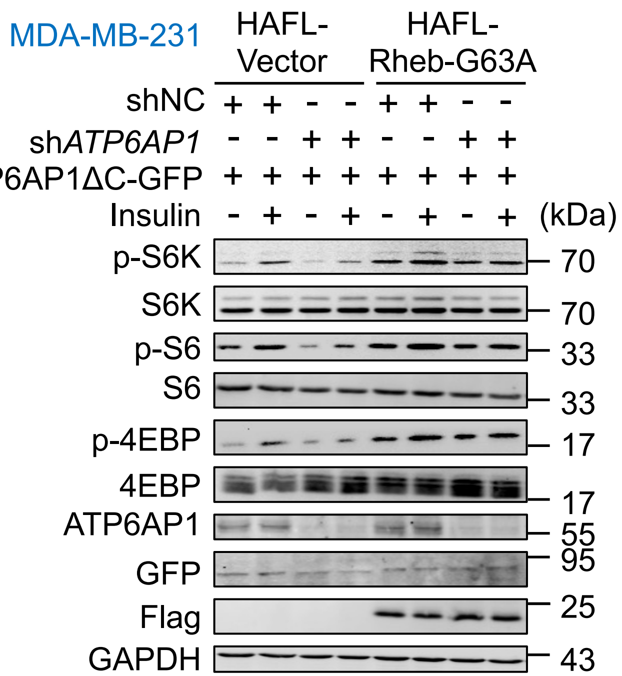

g

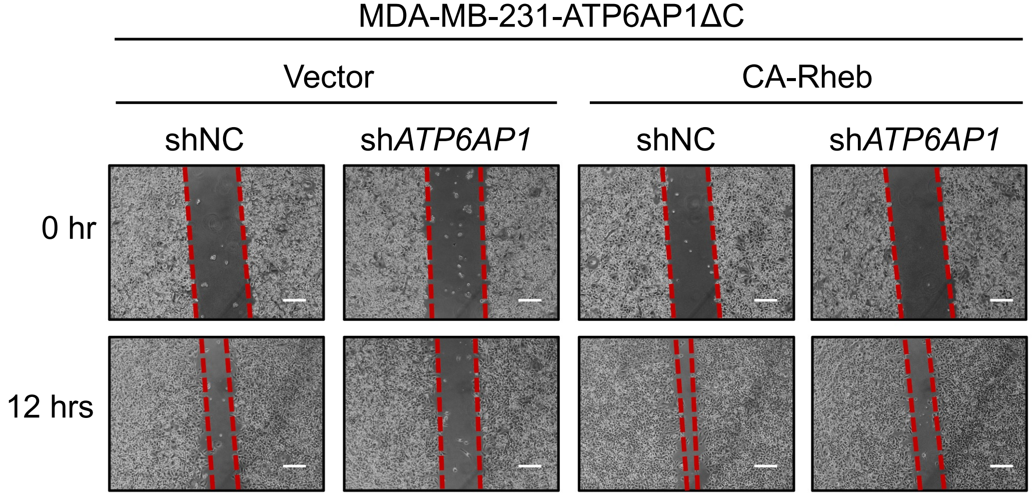

h

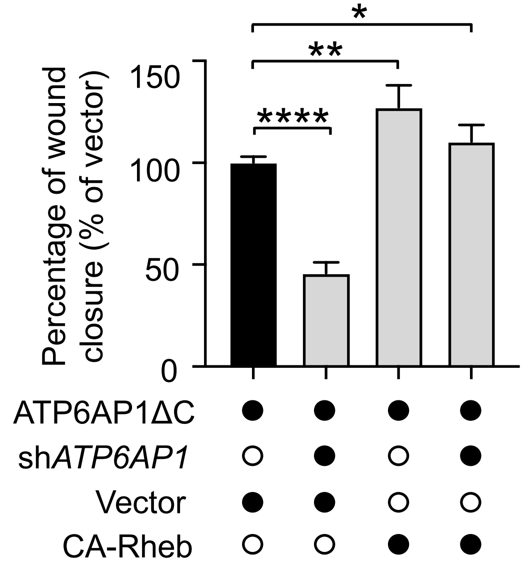

i

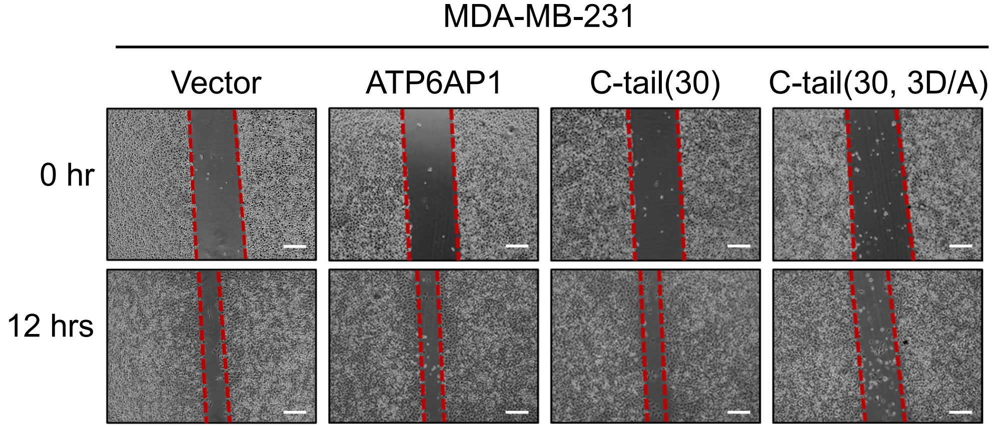

j

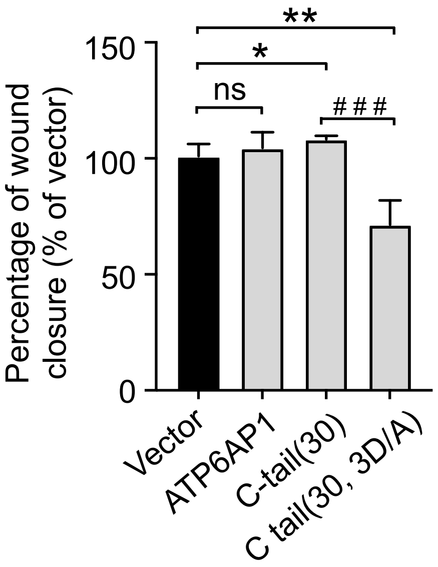

Supplementary information, Fig. S8. ATP6AP1 C-tail loss hampers Rheb activation and expressing C-tail (30, 3D/A) inhibits cancer cell growth and migration

a-c, MDA-MB-231 cells stably expressing the *ATP6AP1*-targeting shRNA along with SFB-tagged full-length or mutant ATP6AP1 were serum starved for 16 hours and then stimulated with insulin (0.9 μM) for 15 minutes before immunoblotting. Intensity values of (a) were obtained using ImageJ. p-S6K (b) and p-S6 (c) signals were normalized to total S6K/S6 as well as GAPDH and graphed as mean ± s.e.m (n=3). Statistical significance was determined using the two-way ANOVA followed by Dunnett's multiple comparisons test, *$p<0.05$, **$p<0.01$, ****$p<0.0001$. shNC with insulin treatment served as a negative control. **d-e**, Cells from (a) were examined in wound healing assays (d). Wound healing data were quantified and presented (e) as mean ± s.e.m (n=3). Vector alone and shNC served as controls. Scale bar: 100 μm. Statistical significance was determined using the two-way ANOVA followed by Dunnett's multiple comparisons test , ***$p<0.001$. **f-h**, MDA-MB-231 cells stably expressing the *ATP6AP1*-targeting shRNA along with GFP-tagged C terminal deletion ATP6AP1 and HA-Flag-tagged Vector or CA-Rheb (constitutively active form of Rheb) were serum starved for 16 hours and then stimulated with insulin (0.9 μM) for 15 minutes before immunoblotting (f) and wound healing assays (g-h). shNC plus vector served as a control. Scale bar: 100 μm (g). Wound healing data were quantified and presented (h) as mean ± s.e.m. (n=3). Statistical significance was determined using the two-way ANOVA followed by Dunnett's multiple comparisons test, *$p<0.05$, **$p<0.01$, ****$p<0.0001$. **i-j**, MD-MBA-231 cells overexpressing SFB-tagged full-length ATP6AP1 or the wildtype/mutant C-tail were examined in wound healing assays (i). Scale bar: 100 μm. The data were presented (j) as mean ± s.e.m., (n=3). *$p<0.05$, **$p<0.01$, ###$p<0.001$. ns, not significant. Vector or C-tail only MD-MBA-231 cells served as negative controls. Statistical significance was determined using the two-tailed Student's *t*-test.
