# Supplementary material for: The rapid proximity labeling system PhastID identifies ATP6AP1 as an unconventional GEF for Rheb
Source: Cell Res. 2024 Mar 6;34(5):355–69. doi: 10.1038/s41422-024-00938-z (PMC11061317; doi:10.1038/s41422-024-00938-z)
Supplement: Supplementary file 9 — Supplementary information, Data S1 [file 41422_2024_938_MOESM9_ESM.pdf]

Supplementary information, Data S1

Supplementary Materials and Methods

Antibodies and other reagents

Reagent Name	Application	Product No.	Supplier
Anti-ATP6AP1	1:200 for WB	sc-81886	Santa Cruz Biotechnology (Dallas, TX, USA)
Anti-Flag	1:5000 for WB	M185-3L	MBL (Beijing, China)
Anti-phospho-S6K1	1:1000 for WB	#9234	Cell Signaling Technology (Danvers, MA, USA)
Anti-S6K1	1:2000 for WB	#9202	
Anti-LAMP1	1:100 for IF	#15665	
Anti-phospho-S6	1:1000 for WB	#4858	
Anti-S6	1:1000 for WB	#2317	
Anti-GST	1:2000 for WB	#2622	
Anti-TPP1	1:2000 for WB	#14667	
Anti-Rheb	1:1000 for WB	#13879	
Anti-mTOR	1:200 for IF	#2983	
Anti-TSC2	1:1000 for WB	#4308	
Anti-Flag	1:800 for IF	#14793	
Anti-GFP	1:1000 for WB	ab32141	abcam (Waltham, MA, USA)
Anti-HA	1:3000 for WB	H6908	Sigma-Aldrich (Louis, MO, USA)

Anti-GAPDH	1:2000 for WB	P30008M	Abmart (Shanghai, China)
Alexa Fluor™ 555 Donkey anti-Mouse IgG		A-31570	Invitrogen (Carlsbad, CA, USA)
Alexa Fluor™ 488 Donkey anti-Rabbit IgG		A-21206	
Streptavidin-FITC	1:1000 for IF	434311	
Streptavidin Protein DyLight™ 680	1:5000 for WB	21848	
IRDye® 800CW Goat anti-Rabbit IgG	1:5000 for WB	926-32211	LI-COR (Lincoln, NE, USA)
IRDye® 680RD Goat anti-Mouse IgG	1:5000 for WB	926-68070	
Immobilized γ-Amino-hexyl-GTP	GTP binding assay	AC-117L	Jena Bioscience (Jena, Germany)
LysoSensor™ Green DND-189	Lysosomal labeling	40767ES50	YEASEN (Shanghai, China)
LysoTracker Red DND-99	Lysosomal labeling	40739ES50	
Pierce™ Streptavidin Magnetic Beads	Streptavidin pull-down	88816	ThermoFisher (Waltham, MA, USA)
Anti-DYKDDDDK (Flag) G1 Affinity Resin	Flag-IP	L00432-5	GeneScript (Nanjing, China)
Glutathione Sepharose 4B	GST pull-down	GE17-0756-01	Cytiva (Sao Paulo, Brazil)

Cell lysis, fractionation, and streptavidin bead pull-down

For cell lysis, cells were resuspended in RIPA buffer (50 mM Tris-HCl, pH 7.4, 150 mM NaCl, 1% Triton X-100, 1% Sodium deoxycholate, 0.1% SDS, 1 mM EDTA, plus 100 mM PMSF and 1% protease inhibitor cocktail (Bimake, B14001)) and incubated on ice for 20 min followed by sonication using the SONICS UibraCell™ VCX150PB sonicator (150 watt, Ampl 25%, 2 s per cycle and 6 s per interval for 10 cycles). The lysates were then centrifuged at 16,000× *g* at 4 °C for 15 min to collect the supernatant for additional processing as needed.

For cell fractionation, 2~5 × 10^7 cells were resuspended in 1 mL Cyto buffer (10 mM HEPES, pH 7.9, 1.5 mM $MgCl_2$, 10 mM KCl, 0.5% NP-40, 1% protease inhibitor cocktail (Bimake, B14001)) for 10 min on ice and then centrifuged (~4,600× *g*) at 4 °C for 10 min to collect the supernatant (cytosolic fraction). The pellet was resuspended in 1 mL RIPA buffer plus 1% protease inhibitor cocktail and incubated on ice for 30 min followed by centrifugation at ~14,000× *g* for 10 min at 4 °C to collect the supernatant (nuclear fraction).

Cell lysates or fractions were incubated with pre-equilibrated streptavidin magnetic beads (Pierce, 88816) on a rotation wheel (Kylin-Bell Lab Instruments) at 4 °C for 2 hours, and then washed successively with 2% SDS, RIPA buffer, 1× TBST, and 1× TBS (twice) on a vortex mixer (VtexMixer VX200, Labnet) at room temperature (1 min/wash). The washed beads were either resuspended in 1× Loading buffer (#FD006, Fude Biological Technology) and incubated at 95 °C for 10 min to collect the supernatant, or in-gel digested for MS analysis as described below.

MS data acquisition, processing, and analysis

TRF1 MS data files (Thermo.raw) and Rheb MS data files (AB SCIEX.wiff) were run by MaxQuant_1.6.2.10[1] and searched against the UniProt human 192283 database (downloaded on 07/09/2020). Parameters include standard contaminants, variable modifications of oxidation (methionine), acetylation (N-terminal), lysine biotinylation, and fixed modification of carbamidomethyl cysteine. Two mis-cleavages of trypsin digestion were allowed. Label-free quantification was used and the iBAQ method[2] was adopted for data processing. The data were screened to remove reverse, potential contaminants, and proteins only identified by site. Proteins with < 2 unique peptides in three repeats were also omitted.

Heatmaps were generated using the R-heatmap package (version 1.0.12). FOT values were normalized to the abundance of the enriched proteins in the particular experiment[3]. FOT is defined as the iBAQ of a given protein (m) divided by the summation of iBAQ of all the identified proteins in one replicate MS test for a particular biotin ligase (n).

$$FOT_{m,n} = \frac{iBAQ_{m,n}}{\sum iBAQ_n}$$

The average of non-zero FOTs for a given protein in three repeats represents its final relative abundance. Fold change was defined as the FOT value in each biotin ligase group divided by the value in the ligase alone control group. The list of candidate proteins was finalized after removal of common contaminant proteins such as keratin, ACACA, ACACB, MCCA, PCCA, and PYC. The Receiver Operating Characteristic (ROC) analysis was performed as described previously[4,5]. A true positive list containing telomere-associated proteins and a false positive list including histone proteins and

nuclear pore proteins were generated based on databases (UniProt, http://www.uniprot.org/uniprot/; Gene Ontology, http://geneontology.org/; BioGrid, http://thebiogrid.org/) and published reports. Statistical analysis and scatterplots were made using GraphPad Prism 9.

To map proximity interactomes, the identified candidates were divided based on published data as well as biological processes, and cellular components using STRING (https://www.string-db.org/)[6], UniProt (https://www.uniprot.org/uniprot/). Proteins involved in multiple pathways or complexes were assigned to the best-known target baits. The networks of known interactors for mTOR and Rheb were established using the molecular complex detection (MCODE) algorithm from Metascape (https://metascape.org/) and BioGRID database, respectively. For subcellular localization and membrane-localized proteins, candidates were classified according to enrichment analysis by Metascape. Interactions between the candidate proteins were mapped using Metascape PPI network including physical and experimental evidence from STRING, BioGrid, and InAct (https://www.ebi.ac.uk/intact/). Final network results were visualized using Cytoscape (version 3.8.2).

Gene Ontology analysis was carried out using Metascape (https://metascape.org/), with the result combining analysis of GO biological processes, cellular components, and molecular functions. Reactome gene sets, terms with an adjusted (Benjamini-Hochberg) p value < 0.05 were ranked based on fold enrichment values and visualized by GraphPad Prism 9.

Protein pull-down and western blot analysis

Protein pull-down and western blotting were performed as described previously[7]. Briefly, cells were lysed in NETN buffer (40 mM Tris-HCl, 100 mM NaCl, 0.5% NP-40, 1 mM EDTA, 10% Glycerol, 1% protease inhibitor cocktail (Bimake, B14001)) followed by sonication and centrifugation at 4 °C (15,000× g) for 15 min. The supernatant was incubated with anti-Flag G1 Affinity Resin (GeneScript, L00432-5) or Glutathione Sepharose 4B (Cytiva, GE17-0756-01) for 2 hours at 4 °C with gentle agitation. The bound proteins were eluted in 1× Loading buffer (#FD006, Fude Biological Technology) for SDS-PAGE and western blotting. Alternatively, cells were lysed in RIPA buffer (50 mM Tris-HCl, pH 7.4, 150 mM NaCl, 1% Triton X-100, 1% sodium deoxycholate, 0.1% SDS, 1 mM EDTA, plus 100 mM PMSF and 1% protease inhibitor cocktail (Bimake, B14001)) and the lysates were resolved by SDS-PAGE and transferred onto PVDF membranes (Millipore) using the Trans-Blot Transfer System (BioRad). Membranes were blocked in 5% BSA before incubation with appropriate antibodies. The membranes were then scanned using the Odyssey imaging system (LI-COR).

Cell staining assays

Immunostaining (IF) assays were carried out as previously described[7]. Briefly, cells grown on glass coverslips were fixed in 4% paraformaldehyde and permeabilized with buffer containing 0.5% Triton X-100, 20 mM HEPES, 50 mM NaCl, 3 mM $MgCl_2$, and 300 mM Sucrose. The coverslips were blocked in blocking buffer (0.2% BSA and 5%

goat serum in PBS) and incubated with primary and secondary antibodies as appropriate. The coverslips were mounted onto glass slides using Vectashield with DAPI (H1000, VECTOR) and imaged on a Lecia TSC SP8 STED 3× fluorescence microscope.

For lysosome studies, cells grown on 35-mm glass-bottom dishes (60-80% confluence) were treated with 1 μM LysoSensor Green DND-189 (40767ES50, YEASEN) for 1 h, washed twice with 1× PBS, and then incubated in fresh medium for 30 min. Hoechst 33342 (C1022, Beyotime) (2 μg/mL) was added to the cells just before live-cell imaging on a Lecia TSC SP8 STED 3× fluorescence microscope equipped with a humidified incubation chamber (Zeiss, Incubator PM S1) maintained at 37 °C with 5% CO_2.

BiFC analysis

The BiFC assay was carried out as previously described[8]. Rheb was cloned into pCL-based retroviral vector for N-terminal tagging with YFPn (amino acids 1-155 of Venus YFP). Vector, GBF1 and INF2 were cloned into pCL-based retroviral vector for C-terminal tagging with YFPc (amino acids 156-239)[9]. HeLa cells stably expressing one of the binding partners were established before stable expression of the other partner.

References

1 Tyanova, S., Temu, T. & Cox, J. The MaxQuant computational platform for mass spectrometry-based shotgun proteomics. *Nat. Protoc.* **11**, 2301-2319 (2016).

2 Schwanhausser, B. et al. Global quantification of mammalian gene expression

control. *Nature* **473**, 337-342 (2011).

3 Zhou, Q. et al. A mouse tissue transcription factor atlas. *Nat. Commun.* **8** (2017).

4 Cho, K. F. et al. Proximity labeling in mammalian cells with TurboID and split-TurboID. *Nat. Protoc.* **15**, 3971-3999 (2020).

5 Hung, V. et al. Spatially resolved proteomic mapping in living cells with the engineered peroxidase APEX2. *Nat. Protoc.* **11**, 456-475 (2016).

6 Szklarczyk, D. et al. STRING v11: protein-protein association networks with increased coverage, supporting functional discovery in genome-wide experimental datasets. *Nucleic Acids Res.* **47**, D607-D613 (2019).

7 Liu, Y. et al. Shwachman-Diamond Syndrome Protein SBDS Maintains Human Telomeres by Regulating Telomerase Recruitment. *Cell Rep.* **22**, 1849-1860 (2018).

8 Ma, W., Kim, H. & Songyang, Z. Studying of telomeric protein-protein interactions by Bi-molecular fluorescence complementation (BiFC) and peptide array-based assays. *Methods Mol. Biol.* **735**, 161-171 (2011).

9 Chen, Y. X. et al. Glycerol kinase-like proteins cooperate with Pld6 in regulating sperm mitochondrial sheath formation and male fertility. *Cell Discov* **3** (2017).
